# CD200 is up-regulated in R6/1 transgenic mouse model of Huntington's disease

**DOI:** 10.1371/journal.pone.0224901

**Published:** 2019-12-02

**Authors:** Andrea Comella Bolla, Tony Valente, Andres Miguez, Veronica Brito, Silvia Gines, Carme Solà, Marco Straccia, Josep M. Canals

**Affiliations:** 1 Stem Cells and Regenerative Medicine Laboratory, Production and Validation Center of Advanced Therapies (Creatio), Department of Biomedicine, Faculty of Medicine and Health Science, University of Barcelona, Barcelona, Spain; 2 Neuroscience Institute, University of Barcelona, Barcelona, Spain; 3 August Pi i Sunyer Biomedical Research Institute (IDIBAPS), Barcelona, Spain; 4 Network Center for Biomedical Research in Neurodegenerative Diseases (CIBERNED), Madrid, Spain; 5 Department of Cerebral Ischemia and Neurodegeneration, Institut d’Investigacions Biomèdiques de Barcelona–Consejo Superior de Investigaciones Científicas (IIBB–CSIC), Barcelona, Spain; 6 Department of Biomedicine, Faculty of Medicine and Health Science, University of Barcelona, Barcelona, Spain; Nathan S Kline Institute, UNITED STATES

## Abstract

In Huntington’s disease (HD), striatal medium spiny neurons (MSNs) are particularly sensitive to the presence of a CAG repeat in the huntingtin (*HTT*) gene. However, there are many evidences that cells from the peripheral immune system and central nervous system (CNS) immune cells, namely microglia, play an important role in the etiology and the progression of HD. However, it remains unclear whether MSNs neurodegeneration is mediated by a non-cell autonomous mechanism. The homeostasis in the healthy CNS is maintained by several mechanisms of interaction between all brain cells. Neurons can control microglia activation through several inhibitory mechanisms, such as the CD200–CD200R1 interaction. Due to the complete lack of knowledge about the CD200–CD200R1 system in HD, we determined the temporal patterns of CD200 and CD200R1 expression in the neocortex, hippocampus and striatum in the HD mouse models R6/1 and HdhQ111/7 from pre-symptomatic to manifest stages. In order to explore any alteration in the peripheral immune system, we also studied the levels of expression of CD200 and CD200R1 in whole blood. Although CD200R1 expression was not altered, we observed and increase in CD200 gene expression and protein levels in the brain parenchyma of all the regions we examined, along with HD pathogenesis in R6/1 mice. Interestingly, the expression of CD200 mRNA was also up-regulated in blood following a similar temporal pattern. These results suggest that canonical neuronal–microglial communication through CD200–CD200R1 interaction is not compromised, and CD200 up-regulation in R6/1 brain parenchyma could represent a neurotrophic signal to sustain or extend neuronal function in the latest stages of HD as pro-survival mechanism.

## Introduction

HD is an autosomal dominant genetic disease caused by a CAG repeat expansion over 37 repeats in the *HTT* gene. Expanded CAG repeats are translated into a series of glutamine residues in the N-terminal region of the huntingtin protein producing a pleiotropic cellular impairment [1]. Although not yet well understood, MSNs in the caudate–putamen nuclei are the most severely affected type of cells in HD [2], resulting in the typical motor impairment known as chorea. However, in recent years it has been demonstrated that a broad neuronal alteration occurs in HD patient’s brains. Indeed, HD causes neurodegeneration at a lesser extent also in cortical and hippocampal regions [3–5], which triggers cognitive impairment and psychiatric symptoms that precede motor dysfunction [6]. Although HD is considered a neurodegenerative disease, all cells in the organism are carrying the mutant Htt (mHtt) protein which may, in turn, alter the physiology of these cells. Peripheral immune system dysregulation produces an increased pro-inflammatory cytokine profile in pre-manifest HD patients, monocyte hyper-responsiveness [7] and migration/recruitment deficits [8]. In addition, kynurenine pathway inhibition in blood results in microglial de-activation in a HD mouse model with a reduced synaptic loss [9]. In the post-mortem HD brain, astrocytosis and microgliosis has been observed in caudate and the internal capsule with an increase complement biosynthesis by reactive microglia [10], which has been recently described as an important mechanism for early synaptic loss in Alzheimer’s disease (AD) [11]. Similarly, microglia activation in HD patient brains is detected years before HD clinical manifestation by magnetic resonance imaging (MRI), allowing to predict disease onset and correlating with disease progression [12]. We recently showed that fingolimod (FTY720), a structural analog of sphingosine that act as an immunomodulatory drug for multiple sclerosis (MS), can also reduce astroglial reactivity in R6/1 mice acting through S1P receptor [13]. Hence, the peripheral immune system and specifically primed microglia activation are likely to play a significant role in neurodegeneration during HD pathogenesis as reported elsewhere [14]. Recently, microglial altered physiology has been proposed as a key factor in the etiology of depression [15], suggesting a multicellular approach to study the biology behind depression and alternative therapeutic strategies. Noteworthy, depression is one of the most common manifestations in the early stage of HD [16]. The highest societal burden associated with HD is due to psychiatric symptoms, which prevalence is estimated between 33% and 76% during disease progression in humans [17].

In normal conditions, neurons are constantly communicating with microglia about their status in order to maintain brain homeostasis [18]. Several cell populations communicate their state constantly in order to maintain the system stable [18–20]. Glial cells can sense neuronal activity in a paracrine manner and through cell-to-cell contacts. Microglia are constantly scavenging the brain parenchyma [21], sensing the surrounding environment for neuronal inputs. These inputs can be classified as “On” or “Off” signals depending on the microglial response they can induce [22]. Usually, the lack of “Off” signals determines microglial activation to reestablish brain homeostasis [22], which is a highly dynamic process in the CNS.

A well-known *in vivo* “Off” signaling system is the one between the transmembrane glycoprotein ligand CD200 (also known as OX-2), mainly expressed by neurons and endothelial cells, and its cognate receptor CD200R1 expressed by myeloid lineage cells, mostly microglia in the brain [23]. Some studies have also reported CD200 expression by oligodendrocytes and astrocytes in MS [24–26]. Interestingly, microglial CD200 expression has been reported only in the hippocampus of an excitotoxic kainic mouse model [27]. CD200 and CD200R1 are highly modulated during mouse CNS development [23], with CD200 usually showing a diffuse distribution in brain parenchyma and a higher intensity in grey matter compared to white matter areas, both in mice and humans [23,25]. Human and mouse brain express two isoforms as a product of an SF2/ASF-dependent alternative splicing mechanism of the CD200 mRNA, a full-length CD200 protein (CD200full) and a truncated isoform (CD200tr). Although CD200tr can bind to CD200R1, it does not activate the downstream signaling pathway, acting as physiological antagonist of the CD200full isoform [28,29]. Moreover, the *Mus musculus Cd200r1* gene is translated into one protein while the human gene encodes four protein isoforms, with two of them lacking of transmembrane and cytoplasmic domains being secreted [30]. In activated mouse microglia, the downregulation of CD200R1 gene expression is regulated by CCAAT/enhancer-binding protein β (C/EBPβ) [31], while anti-inflammatory shift of microglia through CD200–CD200R1 is triggered by the signal transducer and activator of transcription 6 (STAT6)/forkhead box p3 (Foxp3) pathway [32].

Neuronal CD200 is a potent immunosuppressive molecule, in fact its decrease or complete absence induces microglial phagocytosis and pro-inflammatory activation [33,34], which has also been observed to impair hippocampal long term potentiation (LTP) [35] and blood–brain barrier permeability [36]. From a therapeutic point of view, the experimental use of CD200R1 agonists has proven its ability to tune down microglial innate immune response and neurotoxic side effects [37,38]. CD200 is also expressed by lymphoid cells in rats [39] and humans as part of the organism immune regulation [40].

Lack of information about neuronal–microglial communication in HD, and specifically about the CD200–CD200R1 system, prompted us to investigate expression of both CD200 and CD200R1 in HD mouse models. Since ovarian hormones can influence the expression of CD200 receptor in inflammatory conditions [41], we decide to perform the first characterization of CD200 system, in an HD context, in male mice only. Here, we examine the temporal patterns of CD200 and CD200R1 expression in neocortex, hippocampus and striatum of two HD mouse models providing further insight into the function of the neuroimmune system in HD. We also assessed the expression of CD200–CD200R1 in FTY720-treated animals, as we recently reported that the chronic treatment of this immunomodulating drug attenuates astrogliosis and prevents dendritic spines loss in the hippocampus of R6/1 mice [13]. We found a broad upregulation of CD200 expression in the R6/1 brain that increased with HD pathogenesis progression. Furthermore, we provide evidence that increased levels of CD200 in peripheral blood mimic with the increased CD200 levels observed in the CNS along HD pathogenesis.

## Materials and methods

### HD mouse models

Male R6/1 transgenic mice expressing the human exon 1 of the mHtt gene under the control of 1 kb of its human promoter [42] and their corresponding wild-type littermates were obtained from the Jackson Laboratory (Bar Harbor, ME, USA) and maintained in a C57BL/6xCBA background. Genotypes were determined by PCR. CAG-repeat length was determined as previously described [1], and our R6/1 mouse colony carried 145 CAG repeats [43]. We also used HdhQ111/7 heterozygous mutant males and wild-type HdhQ7/7 knock-in mice (C57BL/6 background) generated by knocking-in the full-length chimeric human mHtt exon 1:mouse Htt under the endogenous mouse Htt promoter [44]. Mice were housed together in numerical birth order in groups of mixed genotypes, and data were recorded for analysis by microchip mouse number. The animals had access to food and water *ad libitum* in a colony room kept at 19–22°C and 40–60% humidity, under a 12:12 h light/dark cycle. Animals were sacrificed by cervical dislocation and whole blood was quickly recovered after decapitation. Whole blood and brain samples derived from different animals. Analysis of FTY720 effects was performed on brain samples derived from FTY720-treated animals previously described [13]. Experimental procedures were approved by the Animal Experimentation Ethics Committee of the University of Barcelona in compliance with the Spanish (RD 53/2013) and European (2010/63/UE) regulations for the care and use of laboratory animals.

### RNA isolation, retrotranscription and quantitative real-time PCR

Aqueous phase containing total RNA was isolated from whole blood or brain regions using TRI Reagent (T3809, Sigma-Aldrich) following the manufacturer’s protocol. Then total RNA was purified from the aqueous phase with Direct-zol RNA MiniPrep Plus (R2072, Zymo Research). A range of 0.25 to 1 μg of RNA for each condition were reverse transcribed using a PrimeScript RT reagent kit (RR037A, Takara). cDNA was diluted to 5 ng/μL and 2 μL were used to perform quantitative real-time PCR (Q-PCR). CD200 (NM_010818: Mm.PT.56a.31912048) and CD200R1 (NM_021325: Mm.PT.56a.13442088) transcript expression was detected by PrimeTime qPCR Probe Assays (Integrated DNA Technologies). Actb (NM_007393; Mm.PT.39a.22214843), Gapdh (NM_008084; Mm.PT.39a.1), and Rn18s (NR_003278.3; control 18s) were used as reference genes. Q-PCR was carried out with Premix Ex Taq (RR390A, Takara) in 6 μL final volume using a CFX384-C1000 thermal cycler (Bio-Rad Laboratories). Samples were run for 40 cycles (95°C for 5 s, 60°C for 20 s). Relative gene expression values were calculated by the comparative Ct or ΔΔCt method using the Bio-Rad CFX manager software (Bio-Rad Laboratories).

### Total protein extraction and Western blot

Total protein extracts were obtained after organic separation and homogenization of samples in TRI Reagent (T3809, Sigma-Aldrich) following the manufacturer’s protocol. Protein quantification was determined by the Bradford assay (Bio-Rad Laboratories).

Western blot analyses of 20 μg of total protein extracts were performed as described previously [45], incubating with anti-β-actin (1:20.000 for 30 min; sc-7210, Santa Cruz Biotechnology) or anti-CD200 (1:1000 overnight; AF3355, R&D systems) primary antibodies in immunoblot buffer (Tris-buffered saline (TBS) containing 0.05% Tween-20 and 5% non-fat dry milk); after three washes in phosphate-buffered saline (PBS), membranes were incubated for 1 h with 1:5000 donkey anti-rabbit IgG horse-radish peroxidase (HRP)-conjugate (W4011, Promega) or 1:2000 donkey anti-goat IgG HRP-conjugate secondary antibodies (V8051, Promega), respectively. Chemiluminescent detection was performed incubating with Luminata Classico Western HRP Substrate (WBLUC500, Millipore) for 2 min, and Fuji Medical X-Ray Film Super RX-N (47410 19289, Fujifilm) exposure for 1–10 s. We performed Western blot quantification by ImageJ Gel Analysis plug-in on digital acquired films [46]. Data were expressed as the ratio between the band intensity of the protein of interest and that of β-actin. Relative ratio between CD200 immunoreactive bands was also calculated. Representative Western blots with optimal exposure are shown.

### Immunohistochemistry

Immunohistochemical analysis was performed as previously described [13]. Animals were deeply anesthetized with pentobarbital and intracardially perfused with PBS and a 4% paraformaldehyde solution in 0.1 M sodium phosphate. Brains were removed and post-fixed overnight in the same solution, washed three times with PBS, cryoprotected with 30% sucrose in PBS and frozen in dry-ice cooled methylbutane (Sigma-Aldrich). Serial coronal sections (30 μm) of the brain were obtained using a Microm cryostate and collected in PBS as free-floating sections. The tissue was first incubated with a blocking solution containing PBS, 0.3% Triton X-100, and 5% normal goat serum (Pierce Biotechnology), for 2 h at room temperature. Brain sections were then incubated overnight with shaking at 4°C with the following primary antibodies diluted in the blocking solution: goat polyclonal anti-CD200 (1:200; R&D Systems) and mouse monoclonal anti-NeuN (1:100; Merck). After three washes with PBS, the tissue was incubated for 1 h 30 min at room temperature with specific fluorescent secondary antibodies: Cy2 donkey anti-goat (1:500) and Cy3 donkey anti-mouse (1:500) (Jackson ImmunoResearch). No signal was detected in control sections incubated in the absence of primary antibodies. Images at 10× and 40× magnification were acquired with a Leica SP5 confocal laser scanning microscope (Leica Microsystems).

### Sampling and statistics

Data were analyzed using GraphPad Prism version 6.0c for Mac, GraphPad Software, La Jolla, CA, USA, www.graphpad.com. Outliers were identified through column analysis using a GraphPad integrated package. For the sample data reported here outliers were excluded and only biological replicas were considered. In CNS, the different proteins and the different genes were analyzed in the same set of samples. Samples were tested for normality, using D’Agostino- Pearson omnibus and Shapiro-Wilk normality tests, and equality of variance. Mann–Whitney *U*-test was performed for comparing samples that resulted having significantly different variances when an *F*-test was computed. Multiple *t*-tests were performed to test independent observations between two biological groups. A *p*-value < 0.05 was considered to be statistically significant.

## Results

### CD200, but not CD200R1, gene expression is induced in R6/1 hippocampus and striatum concomitantly with motor symptoms’ appearance

As CD200 protects from inflammation-mediated neurodegeneration [24] and has been recently shown to promote neuronal survival [47], we examined the expression of both CD200 and its receptor CD200R1 during HD pathogenesis in the telencephalon of R6/1 mice. The CNS regions mainly affected in R6/1 mice (i.e., the neocortex, hippocampus, and striatum) were analyzed by Q-PCR at pre-manifest stages of HD (12 weeks), when the HD motor and cognitive phenotype is evident (20 weeks), and at the latest disease stages (30 weeks).

Expression of the CD200 receptor, CD200R1, appeared unaltered in any of the regions analyzed at any time points between genotypes (data not shown). Whereas total CD200 transcript levels were significantly increased in the hippocampus and striatum at all time points, but not in the neocortex (Table 1).

**Table 1:** Time course of total CD200 gene expression in the neocortex, hippocampus, and striatum of R6/1 mice.

CD200 gene expression		Wild-type			R6/1			Statistic	
Area	Age (weeks)	Mean	SD	*n*	Mean	SD	*n*	*p*-value	Result
**Neocortex**	**12**	0.83	0.09	3	1.14	0.36	3	0.227	**=**
	**20**	0.83	0.07	4	0.91	0.27	4	0.604	**=**
	**30**	0.73	0.20	5	0.88	0.33	5	0.392	**=**
**Hippocampus**	**12**	0.65	0.09	6	0.94	0.22	6	0.013	↑
	**20**	0.74	0.09	6	1.12	0.16	6	<0.001	↑
	**30**	0.70	0.12	6	0.96	0.16	6	0.008	↑
**Striatum**	**12**	0.58	0.10	5	1.10	0.13	5	<0.001	↑
	**20**	0.39	0.23	6	1.40	0.99	6	0.035	↑
	**30**	0.63	0.08	5	0.93	0.27	5	0.044	↑

Mean values and SD are relative quantity values after CD200 mRNA normalization the with housekeeping genes Actb, Gapdh, and Rn18S. Multiple *t*-tests are computed to compare wild-type versus R6/1 mice at different time points, assuming that populations do not have the same distribution. Statistical significance (*p*-value) has been corrected for multiple comparisons using the Holm–Sidak method. SD = standard deviation; *n* = number of brain regions from different, non-sibling animals. “= “means no significant differences;”↑” means upregulation.

In MS, CD200 can be expressed by activated astrocytes in the human [25] and mouse [24] CNS. Since we have recently shown that immunomodulating drug FTY720 can attenuate astrocytic activation in R6/1 mice [13], we investigated whether FTY720 chronic treatment in R6/1 mice may restore hippocampal and striatal CD200 gene expression. Interestingly, CD200 and CD200R1 mRNA levels were unmodified by FTY720 treatment in hippocampus and striatum in R6/1 mice (data not shown).

### CD200 protein levels are elevated in late symptomatic stages of telencephalic regions in R6/1 mice

As the CD200–CD200R1 glycoprotein system has functional relevance at protein level triggering intercellular communication, we used samples prepared from neocortex, hippocampus and striatum from wild-type and R6/1 mice to analyze the expression CD200 and CD200R1 proteins by Western blot.

We studied samples from mice 8, 12, 20 and 30 weeks of age, spanning from asymptomatic to latest disease stages, and we found no differences in any region or time point for CD200R1 protein levels between wild-type and R6/1 mice (data not shown), supporting the Q-PCR results.

**Fig 1:**
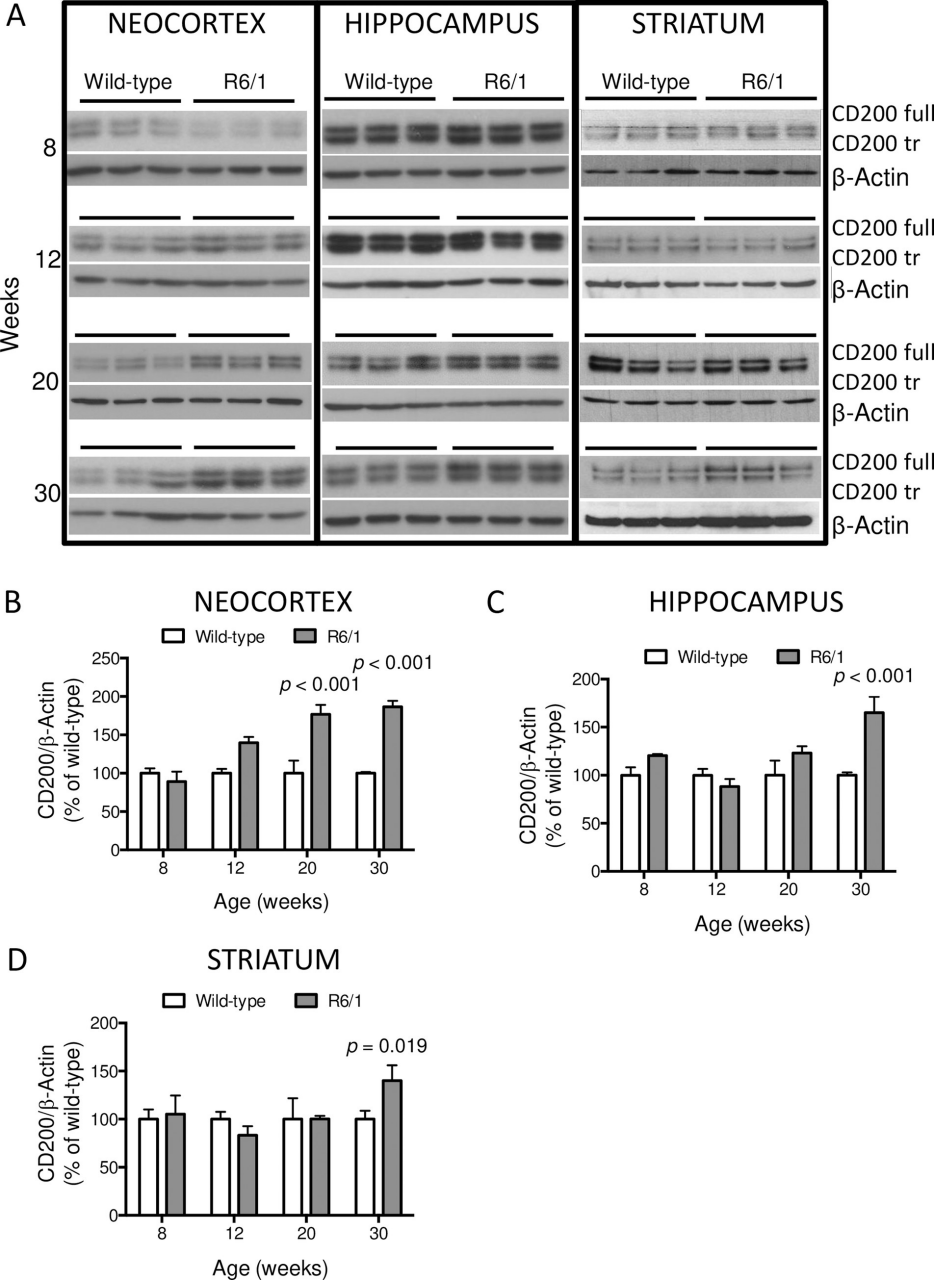
Expression of CD200 protein isoforms in R6/1 mouse brains at 8, 12, 20, and 30 weeks of age. (A) Neocortex, hippocampus and striatum were analyzed by Western blot and CD200 protein levels detected using β-actin as loading control. Both isoforms, CD200full and the truncated CD200tr, are observed in all brain regions under analysis. Quantification of total CD200 protein (both isoforms) was measured in control (wt) and R6/1 mice: (B) in neocortex at 8 (wt = 6; R6/1 = 6), 12 (wt = 3; R6/1 = 3), 20 (wt = 3; R6/1 = 3) and 30 (wt = 3; R6/1 = 3) weeks of age; (C) in hippocampus at 8 (wt = 6; R6/1 = 6), 12 (wt = 5; R6/1 = 5), 20 (wt = 3; R6/1 = 3) and 30 (wt = 6; R6/1 = 6) weeks of age; and (D) in striatum at 8 (wt = 4; R6/1 = 5), 12 (wt = 3; R6/1 = 3), 20 (wt = 3; R6/1 = 3) and 30 (wt = 8; R6/1 = 8) weeks of age. Data are shown as percentage of immunosignal ratio between CD200 and β-actin in R6/1 relative to their respective wild-type mice. Bars represent the means ± standard error of the means (SEM) of 3–8 animals. *p*-Values are the result of the multiple *t*-tests of the R6/1 genotype versus the respective wild-type group.

Western blot analysis for CD200 protein (Fig 1A) detected two immunoreactive signals of about 48 kDa and 45 kDa in all samples, which correspond to the CD200full and truncated isoforms, respectively [28]. As these two isoforms have counteracting functions, we first analyzed their ratio. However, there was no alteration in the ratio of the CD200 full/CD200 truncated isoforms in R6/1 mouse brain regions compared to the wild-type at any time point (data not shown). Hence, we quantified both immunoreactive signals together as the total protein level normalized against β-actin. We analyzed CD200 proteins from wild-type and R6/1 mice in 3 brain regions: I) the neocortex at 8 (wt = 6; R6/1 = 6), 12 (wt = 3; R6/1 = 3), 20 (wt = 3; R6/1 = 3) and 30 (wt = 3; R6/1 = 3) weeks of age (Fig 1B); II) the hippocampus at 8 (wt = 6; R6/1 = 6), 12 (wt = 5; R6/1 = 5), 20 (wt = 3; R6/1 = 3) and 30 (wt = 6; R6/1 = 6) weeks of age; and III) striatum 8 (wt = 4; R6/1 = 5), 12 (wt = 3; R6/1 = 3), 20 (wt = 3; R6/1 = 3) and 30 (wt = 8; R6/1 = 8) weeks of age. CD200 proteins showed a significant increase in neocortex (*p* < 0,001) (Fig 1B), hippocampus (*p* < 0,001) (Fig 1C), and striatum (*p* = 0,019) (Fig 1D) at symptomatic stages in R6/1 mice.

HD hippocampal and striatal regions showed higher levels of CD200 proteins at 30 weeks only. Whereas CD200 protein was significantly upregulated in neocortical samples of R6/1 mice from 20 weeks onwards (Fig 1A and 1B).

**Fig 2:**
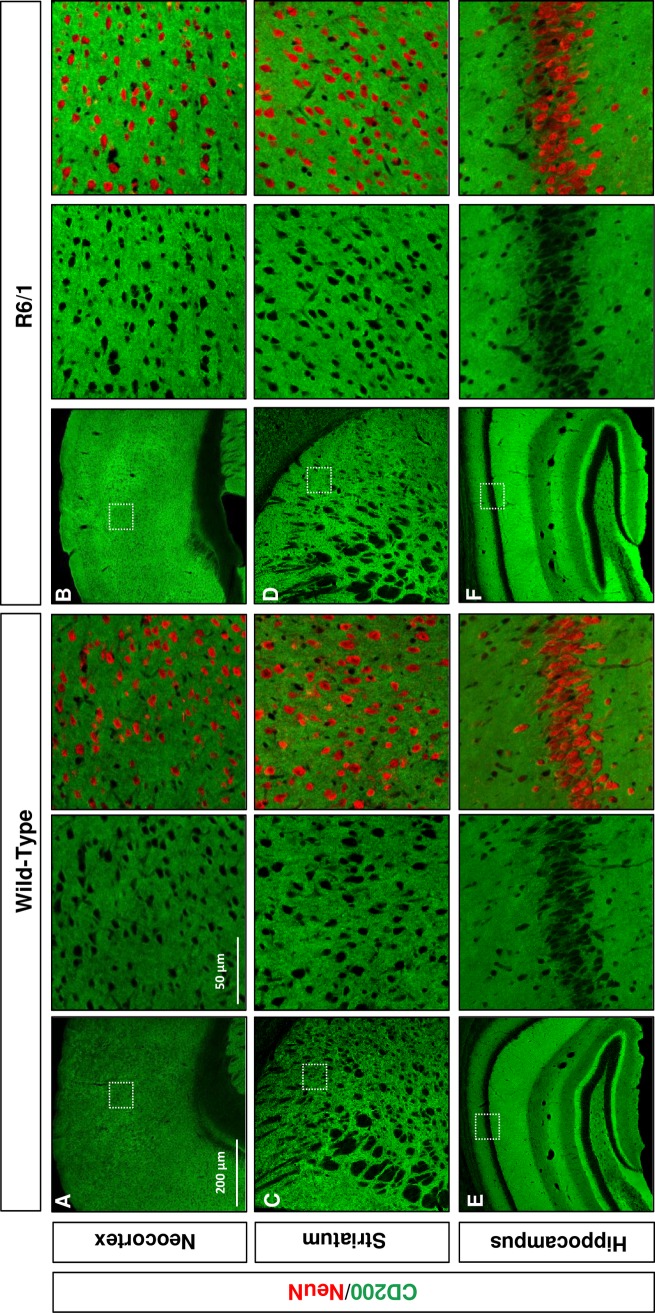
CD200 is increased in R6/1 brain parenchyma. Immunohistochemical comparative analysis of CD200 in wild-type versus R6/1 mouse (A, B) neocortex, (C, D) striatum, and (E, F) hippocampus. Immunolabeling of CD200 is shown in green in representative microphotographs of cryoprotected coronal sections from 20-week-old wild-type and R6/1 mice. Colocalization with NeuN immunolabeling (red) is shown within high magnifications insets for each brain region and genotype. Scale bars = 200 μm and 50 μm. The images shown are representative of four different experiments.

Furthermore, we studied CD200 protein distribution in the neocortex (Fig 2A and 2B), striatum (Fig 2C and 2D) and hippocampus (Fig 2E and 2F) of 25-week-old wild-type and R6/1 mice. CD200 immunostaining displayed strong signal and homogenous distribution in gray matter areas of the neocortex (Fig 2A), striatum (Fig 2C) and hippocampus (Fig 2E) of wild-type mice. The resulting CD200 immunostaining did not allow to determine which type of cell is contributing most to the parenchymal CD200 expression. Although the distribution pattern in brain parenchyma was similar between wild-type and R6/1 mice, the immunohistochemical analysis suggested a qualitative increase in the immunoreactive signal for the CD200 protein in R6/1 mouse brain regions (Fig 2) in supporting of the Western blot results (Fig 1).

### CD200 protein levels in HdhQ111/7 mice

**Fig 3:**
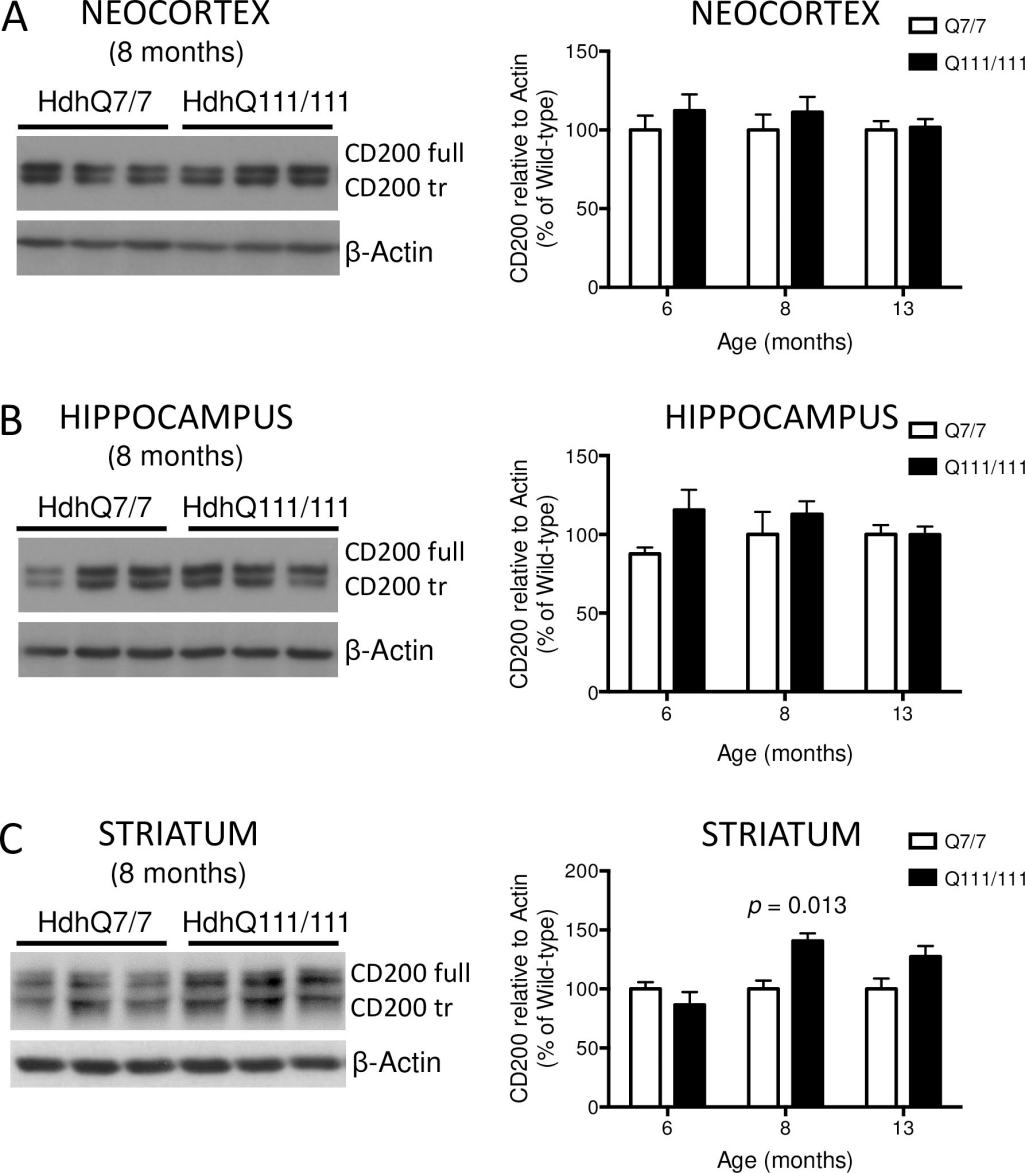
Expression of CD200 protein isoforms in neocortex, hippocampus and striatum of HdhQ7/7 and HdhQ111/7 knock-in mice were analyzed at 6, 8 and 13 months of age by Western blot. CD200full and CD200tr isoforms are observed in the three brain regions. Quantification of CD200 total protein, both isoforms, have been performed (A) in neocortex at 6 (HdhQ7/7 = 13, HdhQ111/7 = 10), 8 (HdhQ7/7 = 7, HdhQ111/7 = 7) and 13 (HdhQ7/7 = 7, HdhQ111/7 = 7) months of age; (B) in hippocampus at 6 (HdhQ7/7 = 8, HdhQ111/7 = 13), 8 (HdhQ7/7 = 7, HdhQ111/7 = 7) and 13 (HdhQ7/7 = 7, HdhQ111/7 = 7) months of age; and (C) in striatum at 6 (HdhQ7/7 = 14, HdhQ111/7 = 10), 8 (HdhQ7/7 = 14, HdhQ111/7 = 10) and 13 (HdhQ7/7 = 7, HdhQ111/7 = 7) months of age. Bars represent the mean ± SEM. *p*-Values are the result of the multiple *t*-tests of the HdhQ111/7 genotype versus the respective HdhQ7/7 group. Representative Western blots for 8 months of age are shown as the percentage of immunosignal ratio between CD200 and β-actin relative to respective HdhQ7/7 brain region.

In order to determine whether the increase of CD200 protein levels was a common feature among genetically different HD mouse models, we also analyzed samples from knock-in HdhQ7/7 and HdhQ111/7 mice by Western blot (Fig 3).

Spanning from 6-month-old (mo) pre-manifest, 8-mo early symptomatic to 13-mo late stages, total protein extract from neocortex (Fig 3A; 6-mo: HdhQ7/7 = 13, HdhQ111/7 = 10; 8-mo: HdhQ7/7 = 7, HdhQ111/7 = 7; and 13-mo: HdhQ7/7 = 7, HdhQ111/7 = 7), hippocampus (Fig 3B; 6-mo: HdhQ7/7 = 8, HdhQ111/7 = 13; 8-mo: HdhQ7/7 = 7, HdhQ111/7 = 7; and 13-mo: HdhQ7/7 = 7, HdhQ111/7 = 7); and striatum (Fig 3C; 6-mo: HdhQ7/7 = 14, HdhQ111/7 = 10; 8-mo: HdhQ7/7 = 14, HdhQ111/7 = 10; and 13-mo: HdhQ7/7 = 7, HdhQ111/7 = 7) were analyzed. CD200 showed mostly no differences between diseased and control mice at protein level. Only at 8 months of age, when motor symptoms begin [48], HdhQ111/7 striatum showed about 30% increase in CD200 (*p* = 0.013) compared to control animals (Fig 3C). However, this significant upregulation of striatal CD200 protein was restored at 13 months of age to control levels. Analysis of CD200R1 protein expression showed the same levels among wild-type and transgenic HD mice (data not shown).

### CD200 transcript levels are increased in peripheral blood of R6/1 mice

**Fig 4:**
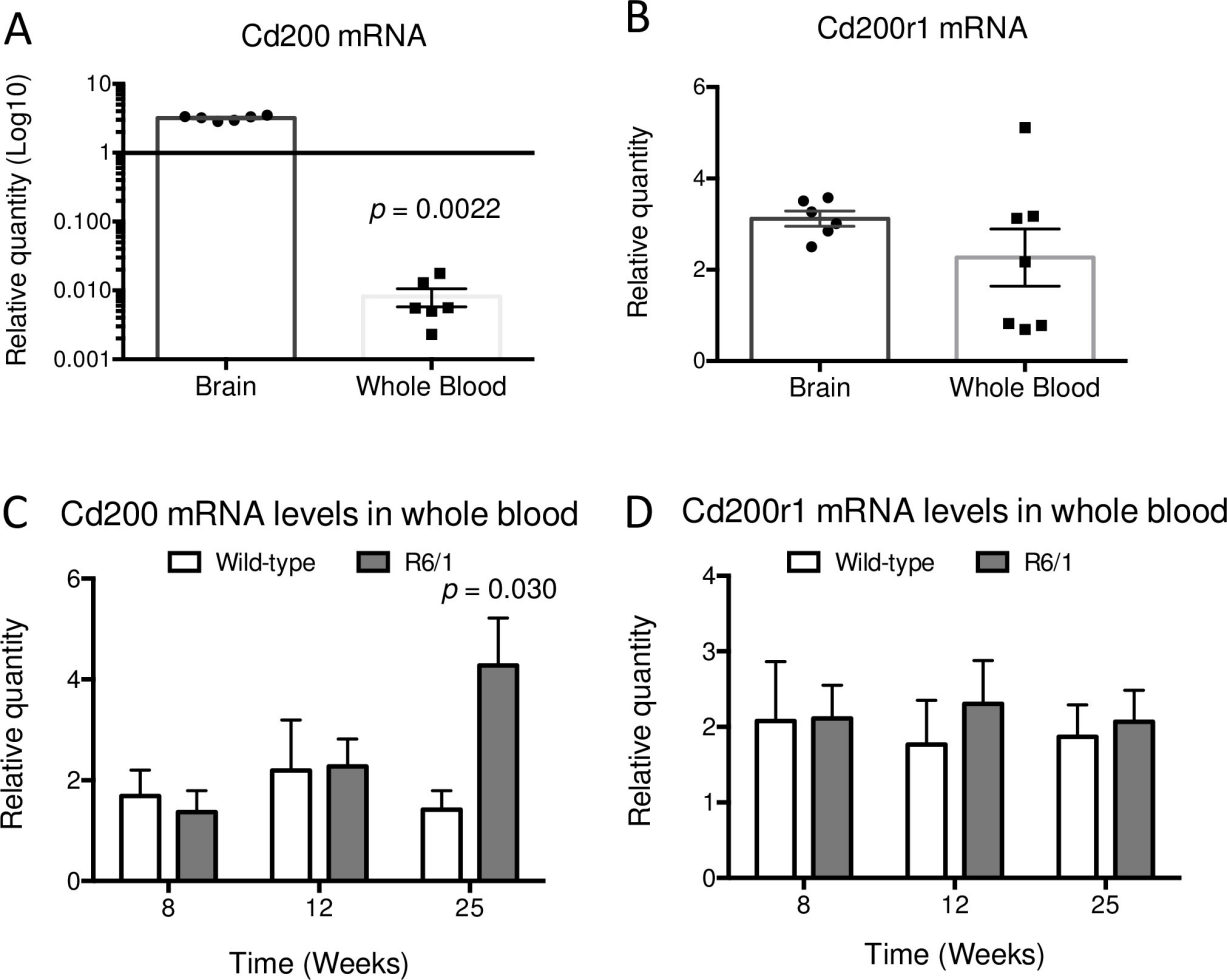
CD200 and CD200R1 gene expression in whole blood of control and R6/1 mice. (A) CD200 and (B) CD200R1 gene expression was analyzed in 25-week-old wild-type mice by qRT-PCR to compare mRNA levels between brain (*n* = 6) and whole blood (*n* = 7), and represented on a scatter dot plot (± SEM). (C) CD200 gene expression was measured in control and R6/1 mouse blood at 8 (wt = 5; R6/1 = 5), 12 (wt = 6; R6/1 = 6) and 25 (wt = 12; R6/1 = 18) weeks of age, and represented on a scatter dot plot (± SEM). (D) CD200R1 gene expression was also analyzed in control and R6/1 mouse blood at 8 (wt = 5; R6/1 = 5), 12 (wt = 7; R6/1 = 5) and 25 (wt = 13; R6/1 = 17) weeks of age. Both time courses are represented by a box plot with minimum and maximum whiskers.

As CD200 is also expressed in B- and T-cells [40,49], we decided to examine CD200 mRNA expression in the whole blood of R6/1 mice along HD pathogenesis. First, we compared CD200 relative mRNA levels between brain (n = 6) and whole blood (n = 7) in wild-type mice. These analyses of CD200–CD200R1 in mouse blood showed that CD200 mRNA levels in the brain are significantly higher than in blood, which was not previously reported. We observed very low CD200 gene expression in peripheral blood compared to the brain (*p* = 0.0022) (Fig 4A). Conversely, CD200R1 gene levels were similar (*p* = 0.23) between brain and whole blood of wild-type mice (Fig 4B).

Next we determined CD200 (Fig 4C) and CD200R1 (Fig 4D) mRNA levels in peripheral blood comparing wild-type and R6/1 mice at pre-symptomatic (8 weeks; wt = 5; R6/1 = 5), prodromal (12 weeks; wt = 6; R6/1 = 6) and at manifest (25 weeks; wt = 12; R6/1 = 18) stages of HD pathogenesis. The CD200R1 mRNA levels showed no difference between genotypes at any stage (Fig 4D). Conversely, CD200 gene expression was higher (*p* = 0.03) in peripheral blood of R6/1 mice at 25 weeks of age when motor and cognitive symptoms are manifested (Fig 4C), mirroring CD200 increased levels in neocortex, striatum and hippocampus.

## Discussion

In healthy brain, neuronal–glial cross-talk is maintained through different signaling pathways, notably the CD200–CD200R1 system. As shown in Table 2, this system is often altered in animal models of physiological aging or neurological disorders (such as MS, Parkinson’s disease, depression, stress, and CNS infections) and in neurological symptoms of peripheral immune system activation. Moreover, it is usually impaired in post-mortem samples of patients with MS, AD, epilepsy and Lewy body-associated dementia (see Table 2 for references). A dramatic reduction in CD200 expression levels is the common observation in most of the studies (Table 2); whereas the expression levels of CD200R1 are altered in both directions. As reported in the literature, a biological interpretation of these results suggests that neuronal CD200 downregulation is a common feature of endangered neurons, which could activate microglia to stop disease progression. This appears to hold true from viral infection models that highlight the evolutionary conserved role of CD200, where its decrease triggers the innate immune response to stop the infection. In this view, neurodegenerative diseases are depend on the same innate immune pathway, chronically activating microglia through CD200 downregulation [50]. On the other hand, CD200R1 levels reflect different states of microglial activation. Furthermore, microglia react to the decrease of neuronal CD200 in a dynamic- and disease-dependent manner, computing an output between pro- and anti-inflammatory activation [51].

**Table 2:** Relevant literature on CD200–CD200R1 mRNA or protein levels in neurodegenerative disorders, neurological sequelae of infections, or experimental peripheral inflammation in human or animal models.

Neurological disorder	CD200	CD200R1	Areas	Endpoint	Model	Reference
**Aging**	Decreased	-	Hippocampus	Protein	*Rattus norvegicus*	[63]
**Aging**	Decreased	-	Hippocampus	mRNA	*Rattus norvegicus*	[64]
**Aging**	Decreased	-	Hippocampus	Protein	*Rattus norvegicus*	[65]
**Aging**	Decreased	-	Hippocampus	mRNA	*Rattus norvegicus*	[66]
**Aging**	Decreased	-	Substantia nigra	mRNA	*Rattus norvegicus*	[67]
**Aging + Postoperative cognitive dysfunction**	Decreased	-	Hippocampus	mRNA	*Rattus norvegicus*	[68]
**Aging + Postoperative cognitive dysfunction**	Decreased	-	Hippocampus	mRNA	*Rattus norvegicus*	[69]
**Aging + Postoperative cognitive dysfunction**	Decreased	Unchanged	Hippocampus	mRNA	*Rattus norvegicus*	[70]
**Alzheimer's disease**	Decreased	Decreased	Hippocampus/Inferior temporal gyrus	mRNA/Protein	*Homo sapiens*	[26]
**Alzheimer's disease with Lewy bodies**	Decreased	-	Temporal/Cingulate cortex	Protein	*Homo sapiens*	[71]
**Chronic intractable epilepsy**	Decreased	Unchanged	Cortex	mRNA/Protein	*Homo sapiens*	[72]
**Dementia with Lewy bodies**	Decreased	-	Temporal cortex	Protein	*Homo sapiens*	[71]
**Influenza A/PR/8/34 (H1N1) virus**	Decreased	-	Hippocampus	mRNA	*Mus musculus*	[73]
**Influenza A/PR/8/34 (H1N1) virus**	Decreased	-	Hippocampus	mRNA	*Mus musculus*	[74]
**Multiple sclerosis**	Decreased	Increased	Brain/Spinal cord	mRNA/Protein	*Mus musculus*	[75]
**Multiple sclerosis**	Decreased	Unchanged	Cortex/Spinal cord	mRNA/Protein	*Homo sapiens*	[76]
**Parkinson's disease**	Decreased	Decreased	Brain	Protein	*Mus musculus*	[77]
**Parkinson's disease**	Decreased	Decreased	Ventral midbrain	Protein	*Mus musculus*	[78]
**Peripheral inflammation**	Decreased	Decreased	Brain	mRNA	*Mus musculus*	[79]
**Peripheral inflammation**	Decreased	Increased	Substantia nigra	mRNA	*Rattus norvegicus*	[37]
**Peripheral inflammation**	Decreased	-	Hippocampus	mRNA	*Mus musculus*	[80]
**Respiratory infection**	Decreased	Increased	Hippocampus/Prefrontal cortex	mRNA	*Sus scrofa*	[81]
**Stress and infection**	Decreased	-	Hippocampus	mRNA	*Rattus norvegicus*	[82]
**Theiler's virus-induced demyelinating disease**	Decreased	Decreased	Spinal cord	mRNA	*Mus musculus*	[83]
**Venezuelan equine encephalitis virus infection**	Decreased	-	Brain	Protein	*Mus musculus*	[84]
**Exitotoxic injury**	Increased	-	Hippocampus	Protein	*Mus musculus*	[27]
***Toxoplasma gondii* encephalitis**	Increased	Increased	Brain	Protein	*Mus musculus*	[52]
**Prion disease**	n.a.	Increased	Hippocampus	mRNA	*Mus musculus*	[85]
**Acute stress**	Unchanged	Decreased	Hypothalamus	mRNA	*Rattus norvegicus*	[86]
**Depression**	Unchanged	Decreased	Hippocampus	mRNA	*Rattus norvegicus*	[87]
**Exitotoxic injury**	Unchanged	-	Hippocampus	mRNA	*Rattus norvegicus*	[88]
***Trypanosoma brucei* infection**	Unchanged	Unchanged	Brain	mRNA	*Mus musculus*	[89]

Literature search was performed using Scopus and the PubTator text-mining tool by PubMed, searching for “CD200” and “Brain”. Articles focused on *in vitro* models, CNS neoplasias, and reviews were excluded.

Worth noting, here we described a clear increase of CD200 levels in the neocortex and striatum of R6/1 mice with no alteration of CD200R1 expression. Together, these results suggest that a non-cell autonomous mechanism in HD differs from other neurodegenerative diseases in this animal model. To the best of our knowledge, only two studies showed a CD200 increase in mouse, one in a toxoplasma-associated encephalitis model [52] and another in an excitotoxicity mouse model [27]. Interestingly, excitotoxicity has been proposed as one of the driving MSNs neurodegeneration forces in HD [53]. In fact, mHtt MSNs are vulnerable to glutamate-triggered excitotoxicity [54] and it is suggested that an altered glutamatergic transmission in the cortico-striatal synapses can be involved in HD neurodegeneration [55]. However, R6/1 mice of 18 weeks of age have been shown to be resistant to excitotoxic insults [56]. Our findings not only suggest that increased levels of CD200 in the neocortex and striatum of R6/1 mice could reinforce the hypothesis of an active excitotoxic process in the cortico-striatal pathway, but also point to a neuroprotective role of CD200 towards neuronal excitotoxicity.

We also observed an increase in CD200 expression in hippocampal regions. Interestingly, CD200 knockout mice showed a long-term potentiation (LTP) impairment due to an altered neuroinflammatory response [35]. Hippocampal LTP is also altered in R6/1 mice [43] and here we showed that their hippocampi express higher levels of CD200 than in control animals. Hippocampal LTP impairment in HD has been associated with spatial and recognition memory deficits [5] and this has been linked to a reduction of brain-derived neurotrophic factor (BDNF) levels [43]. In this context, CD200 upregulation in R6/1 hippocampus can represent a mechanism to counteract neuroimmune response [24,38] and phagocytosis [33], and a likely synaptic pruning [10,57]. On the other hand, CD200 upregulation could also represent a compensatory signal to promote neuronal survival in an advanced HD context, where BDNF is reduced. In fact, normal levels of CD200R1 in R6/1 mouse brains suggest an augmented availability of CD200 ligand that would be able to interact with other receptors. In fact, interaction of neuronal CD200 with the fibroblast growth factor receptor 1 (FGFR1) has been reported [47]. Pankratova et al. described that CD200 is a partial agonist of FGFR1 *in vitro*, which triggers neurites outgrowth in hippocampal neuronal cultures. The interpretation that CD200 increase in the R6/1 mouse brain may act through other receptors that CD200R1 would explain the lack of effect of FTY720 treatment on modulating CD200–CD200R1, since they both could attenuate gliosis through different pathways.

It is worth highlighting the differences between the two HD mouse models we screened, the human mHtt exon-1 R6/1 model and the full-length knock-in HdhQ111/7 model. Although R6/1 animals have shown a broad increase of CD200, HdhQ111/7 mice have shown only a transient upregulation in the striatum. Although we cannot exclude that CD200 can be modulated at older ages in HdhQ111/7 mice, differences between these two models in terms of behavioral and neuropathological symptoms have been reported elsewhere [58,59]. Among these differences, there are no studies describing a classical pro-inflammatory innate immune activation in R6/1 mice, while innate immune activation in YAC128 and R6/2 HD transgenic mice has been described previously [7,60]. In agreement with our results from R6/1 mice, our observations suggests that CD200 upregulation in the neocortex, and later in hippocampus and striatum, could mediate the resilience of this HD mouse model to show a clear pro-inflammatory microgliosis in the diseased parenchyma [24]. In addition, CD200 upregulation in R6/1 mouse blood could also explain why these HD mice do not have a peripheral immune inflammation as shown by other HD mice models [7]. This hypothesis confirms the strong immunosuppressive and immunomodulatory properties of CD200 function, abundantly reported in the literature [61,62]. Further research in HD patients’ bio-specimens or human multicellular stem cell-derived *in vitro* models may allow us to assess the relevance of CD200 in the human pathogenesis of HD.

In conclusion, here we described for the first time the temporal expression pattern of the CD200 and CD200R1 in the CNS and blood of the R6/1 HD model. In this study, we demonstrated that there is a transcriptional upregulation of CD200 from pre-symptomatic stages and an increase of the protein levels detectable only at the symptomatic phase of HD. No alterations of the myeloid receptor CD200R1 have been detected, suggesting that microglia–neuronal cross-talk is not impaired. However, the extra CD200 ligand could function as a neurotrophic signal promoting survival of HD neurons. Hence, we suggest to further investigate CD200 as a possible pro-survival mechanism in HD pathogenesis in the R6/1 mouse model.

## Supporting information

S1 File Original western blots without modifications. (PDF)
