# Supplementary material for: CD200 is up-regulated in R6/1 transgenic mouse model of Huntington's disease
Source: PLoS One. 2019 Dec 2;14(12):e0224901. doi: 10.1371/journal.pone.0224901 (PMC6886799; doi:10.1371/journal.pone.0224901)

MANUSCRIPT NUMBER: PONE-D-19-19201

PLOS ONE

CD200 is up-regulated in R6/1 transgenic mouse model of Huntington's disease

Andrea Comella Bolla[1,2,3,4,¶], Tony Valente[3,5], Andres Miguez[1,2,3,4], Veronica Brito[2,3,6], Silvia Gines[2,3,6], Carme Solà[3,5], Marco Straccia[1,2,3,4,7,¶*] and Josep M. Canals[1,2,3,4*]

[1] Stem Cells and Regenerative Medicine Laboratory, Production and Validation Center of Advanced Therapies (Creatio), Department of Biomedicine, Faculty of Medicine and Health Science, University of Barcelona, Barcelona, Spain

[2]Neuroscience Institute, University of Barcelona, Barcelona, Spain

[3]August Pi i Sunyer Biomedical Research Institute (IDIBAPS), Barcelona, Spain

[4]Network Center for Biomedical Research in Neurodegenerative Diseases (CIBERNED), Spain

[5]Department of Cerebral Ischemia and Neurodegeneration, Institut d'Investigacions Biomèdiques de Barcelona–Consejo Superior de Investigaciones Científicas (IIBB–CSIC), Spain

[6]Department of Biomedicine, Faculty of Medicine and Health Science, University of Barcelona, Barcelona, Spain

[7]Present address: FRESCI, Freelance Scientists, Human Technology Consultants – www.fre-sci.com

[¶]These authors contributed equally to this work.

*Corresponding authors: e-mail: marco.straccia@fre-sci.info (MS); e-mail: jmcanals@ub.edu (JMC)

Figura 1 A: 8 weeks samples page 1/2

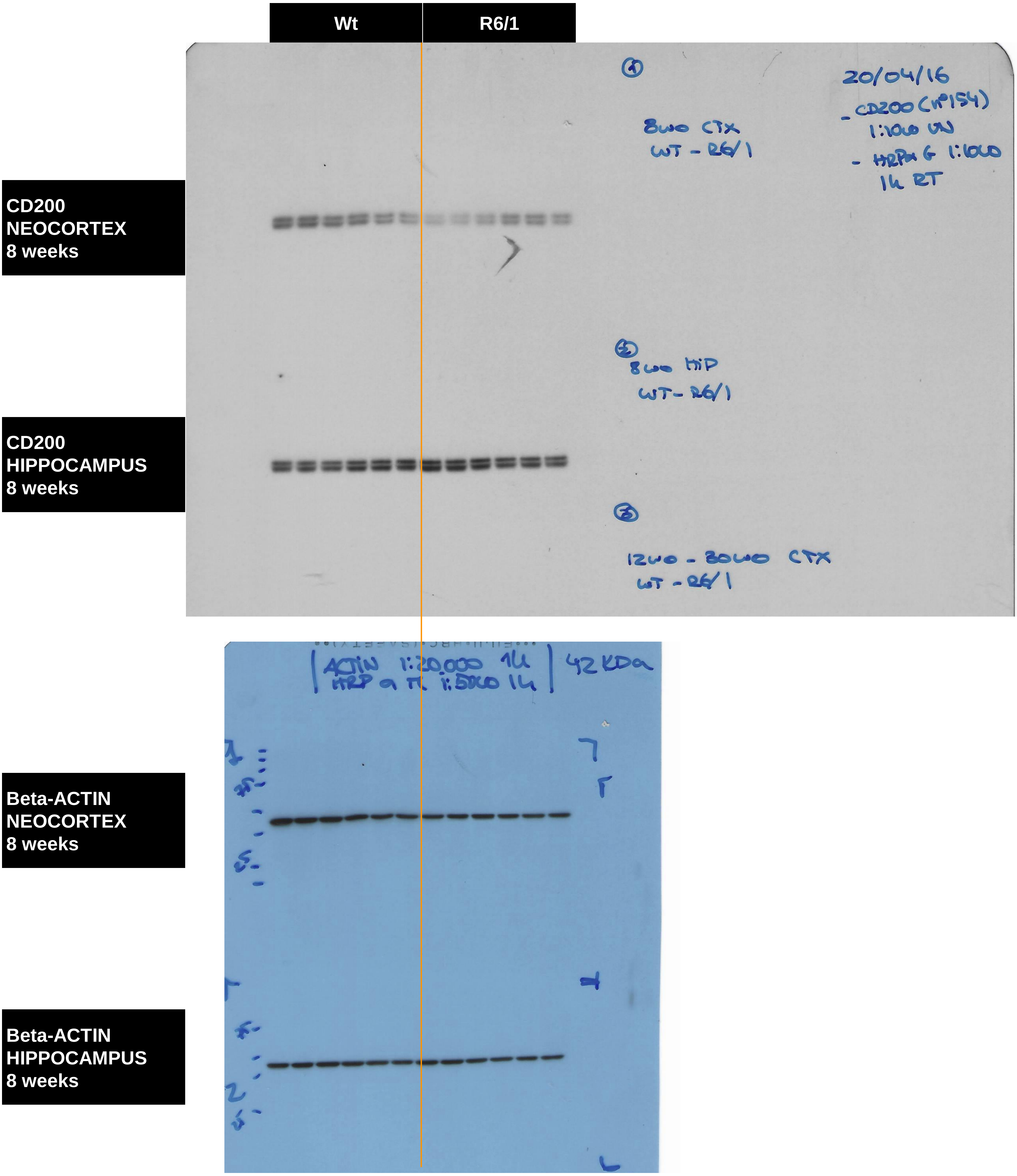

Figura 1 A: 8 weeks samples
page 2/2

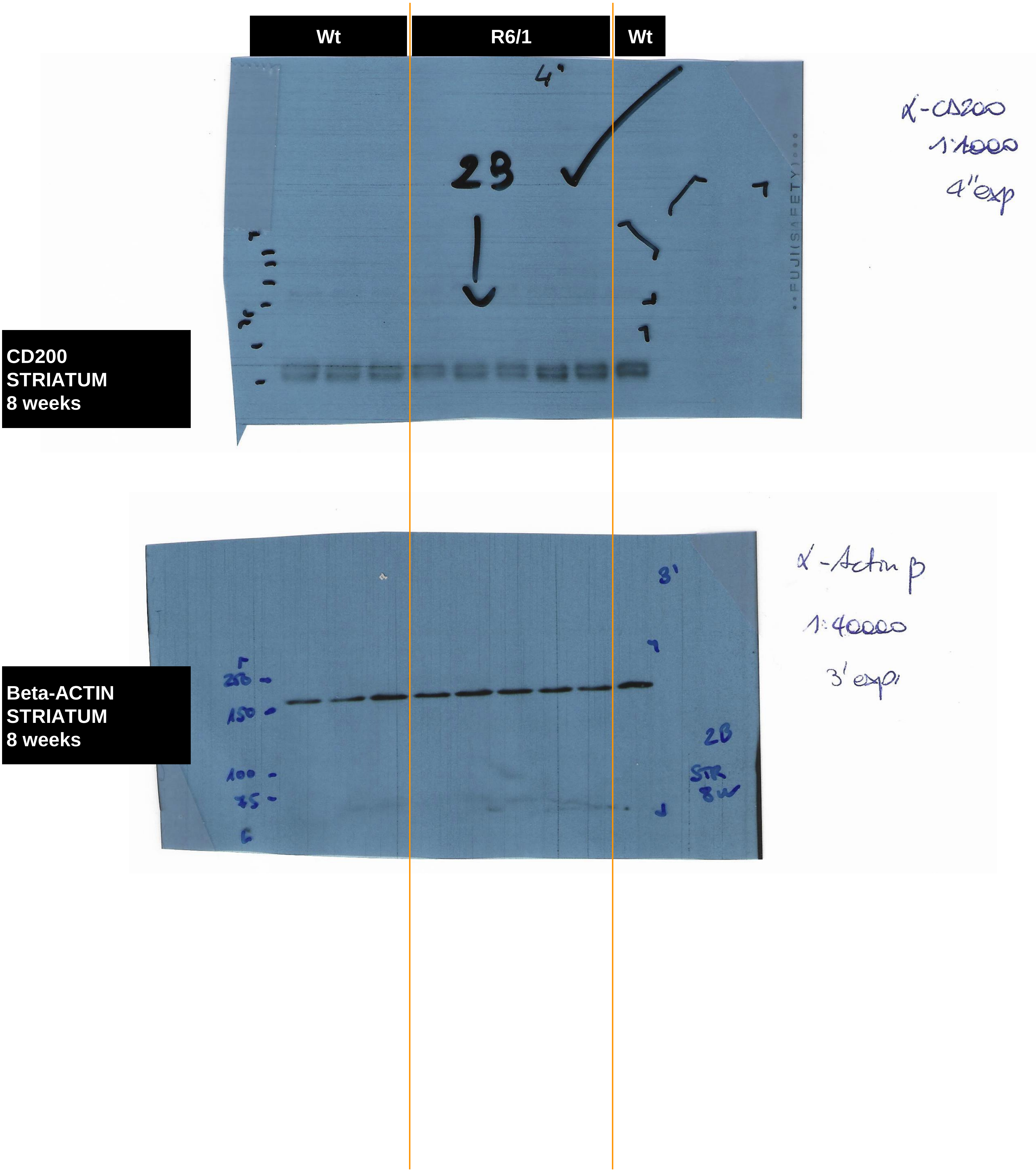

Figura 1 A: 12 weeks samples page 1/2

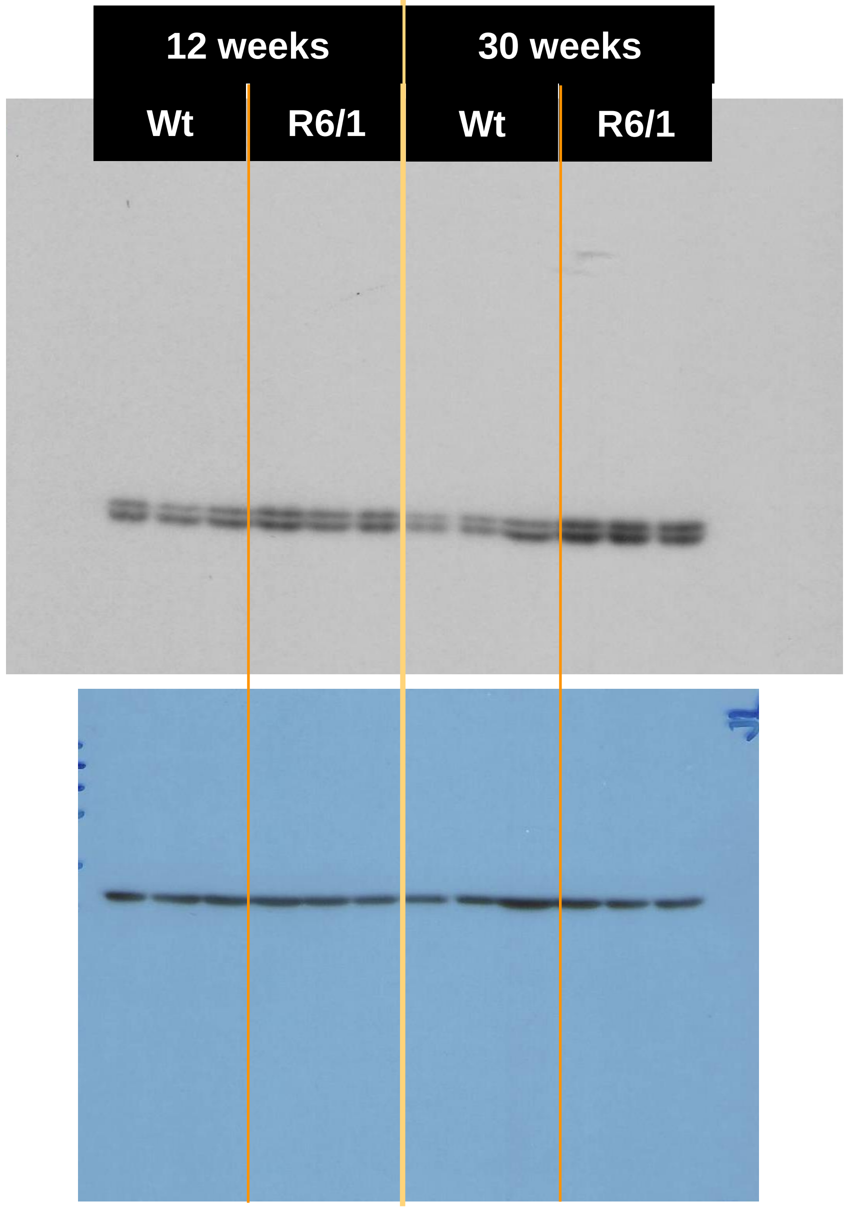

CD200
NEOCORTEX
12 - 30 weeks

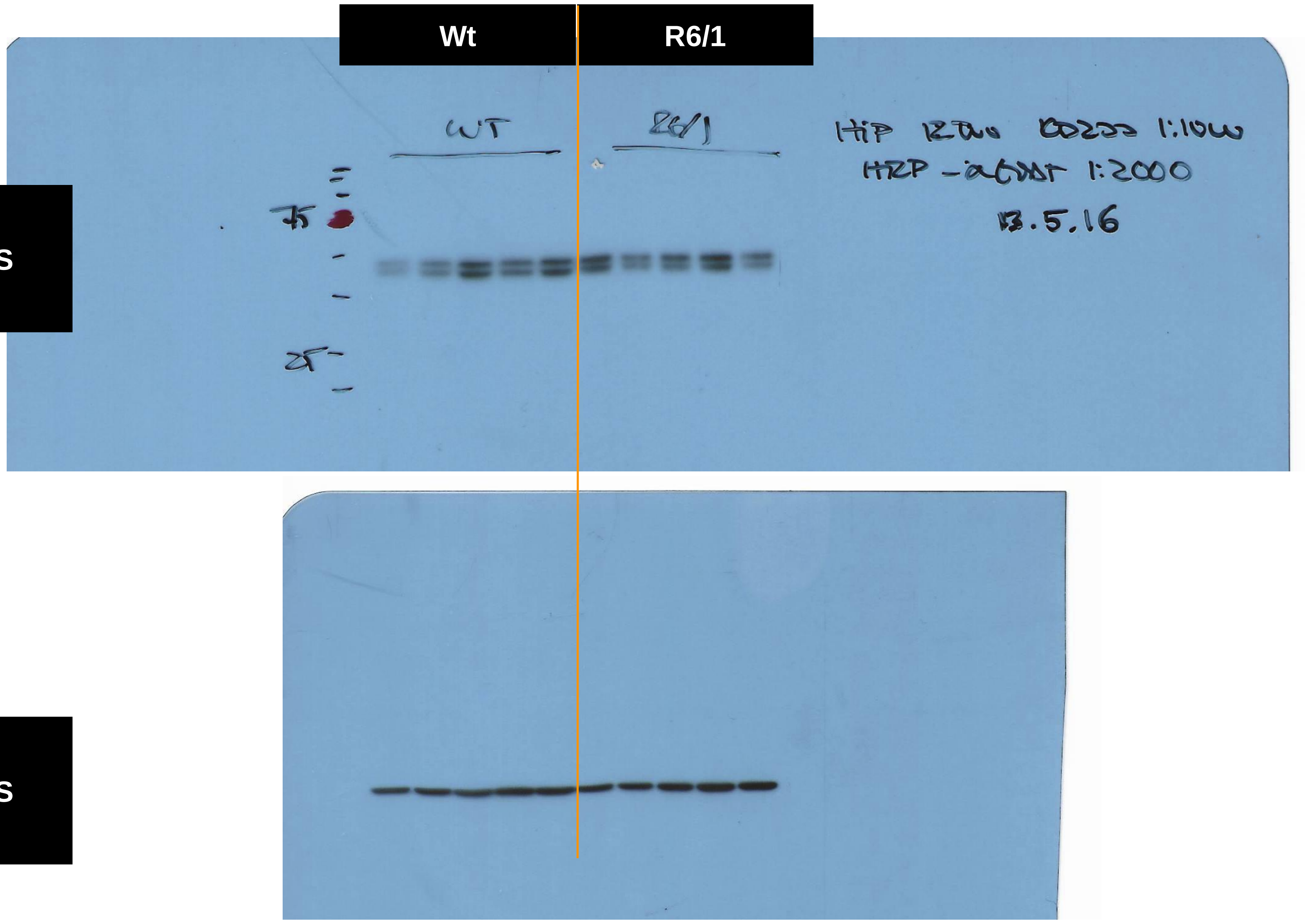

CD200
HIPPOCAMPUS
12 weeks

Beta-ACTIN
HIPPOCAMPUS
12 weeks

Figura 1 A: 12 weeks samples
page 1/2

CD200
STRIATUM
12 weeks

Wt | R6/1

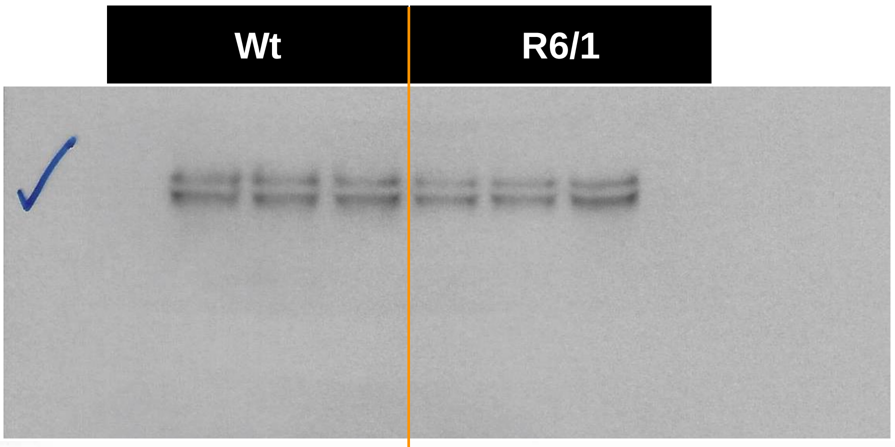

Wt | R6/1

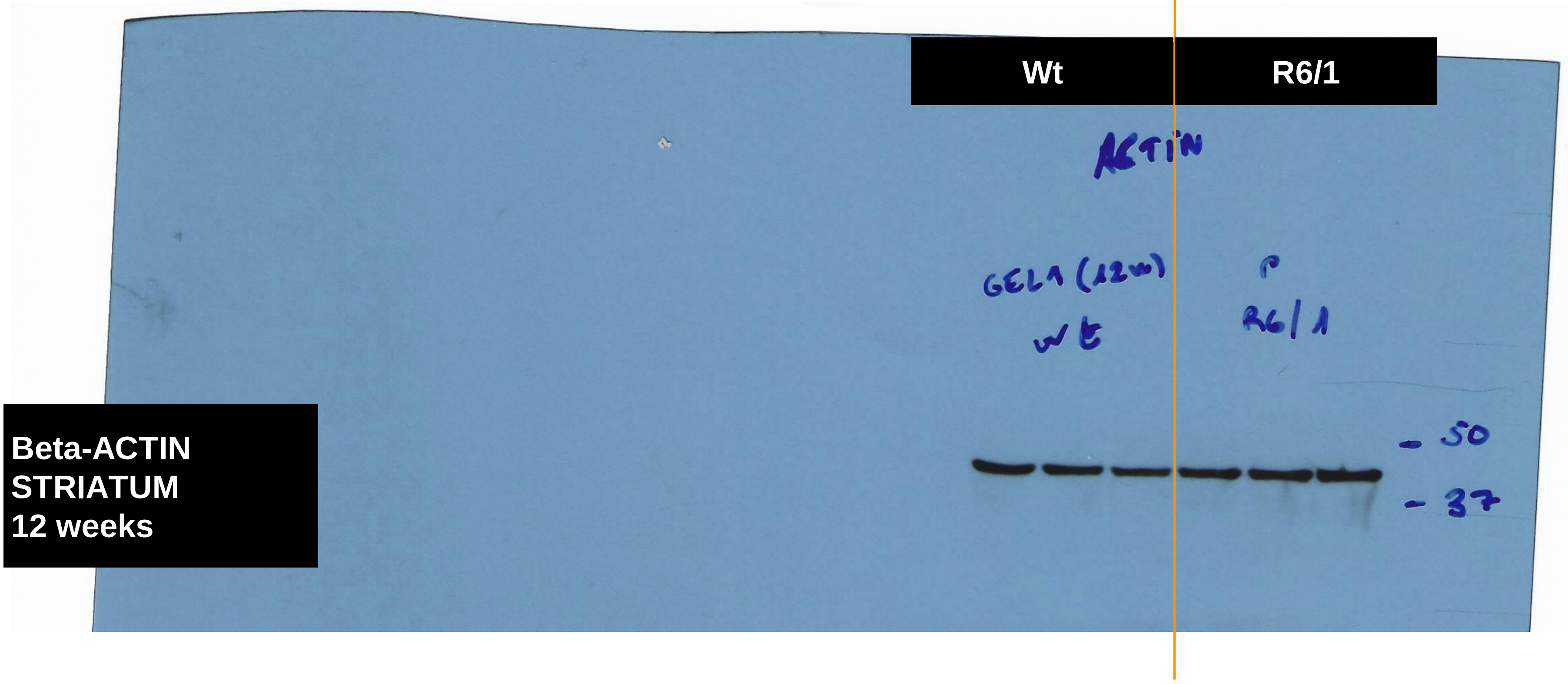

Beta-ACTIN
STRIATUM
12 weeks

Figura 1 A: 20 weeks samples page 1/2

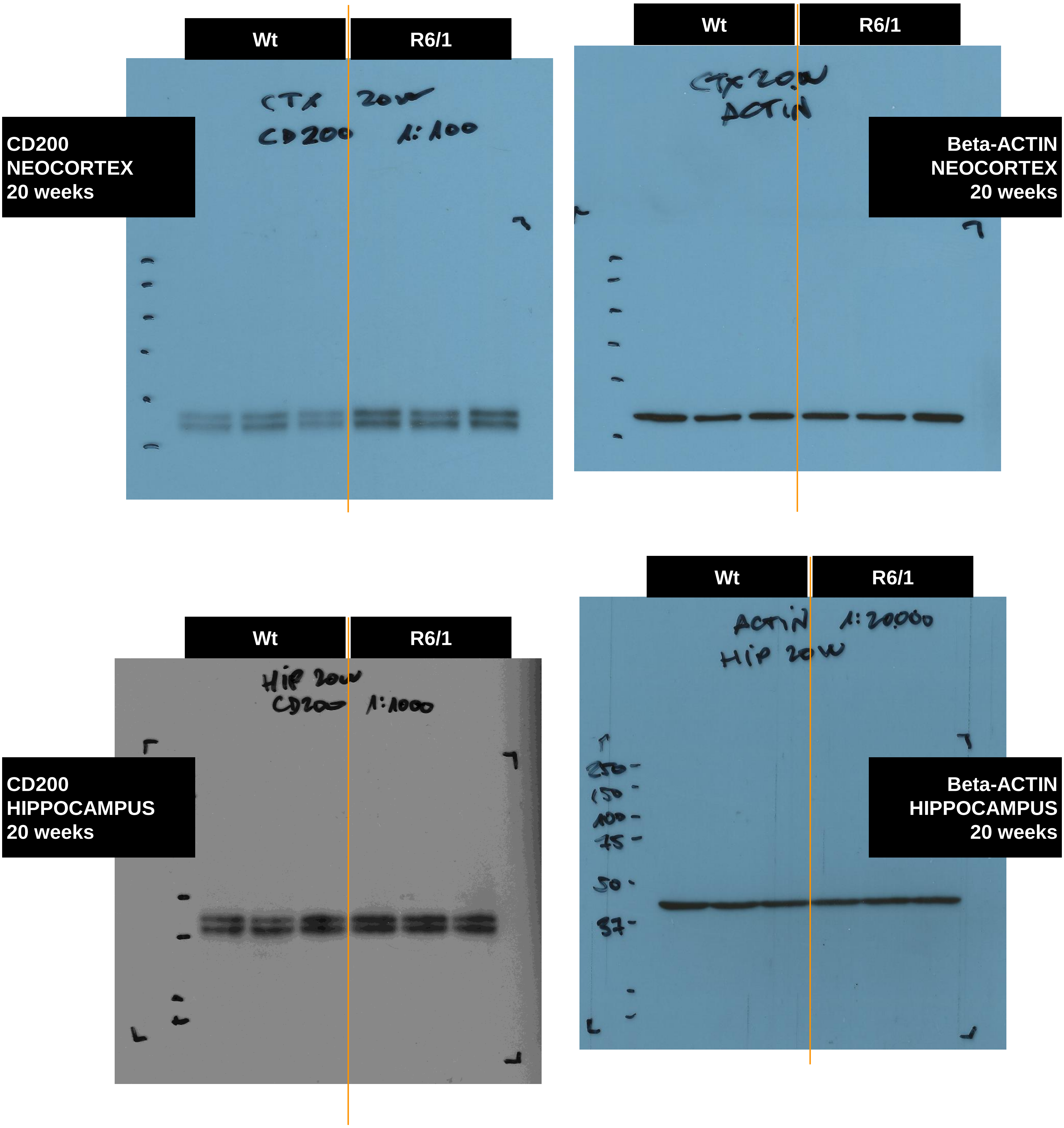

Figura 1 A: 20 weeks samples
page 2/2

Wt	R6/1

Beta-ACTIN
STRIATUM
20 weeks

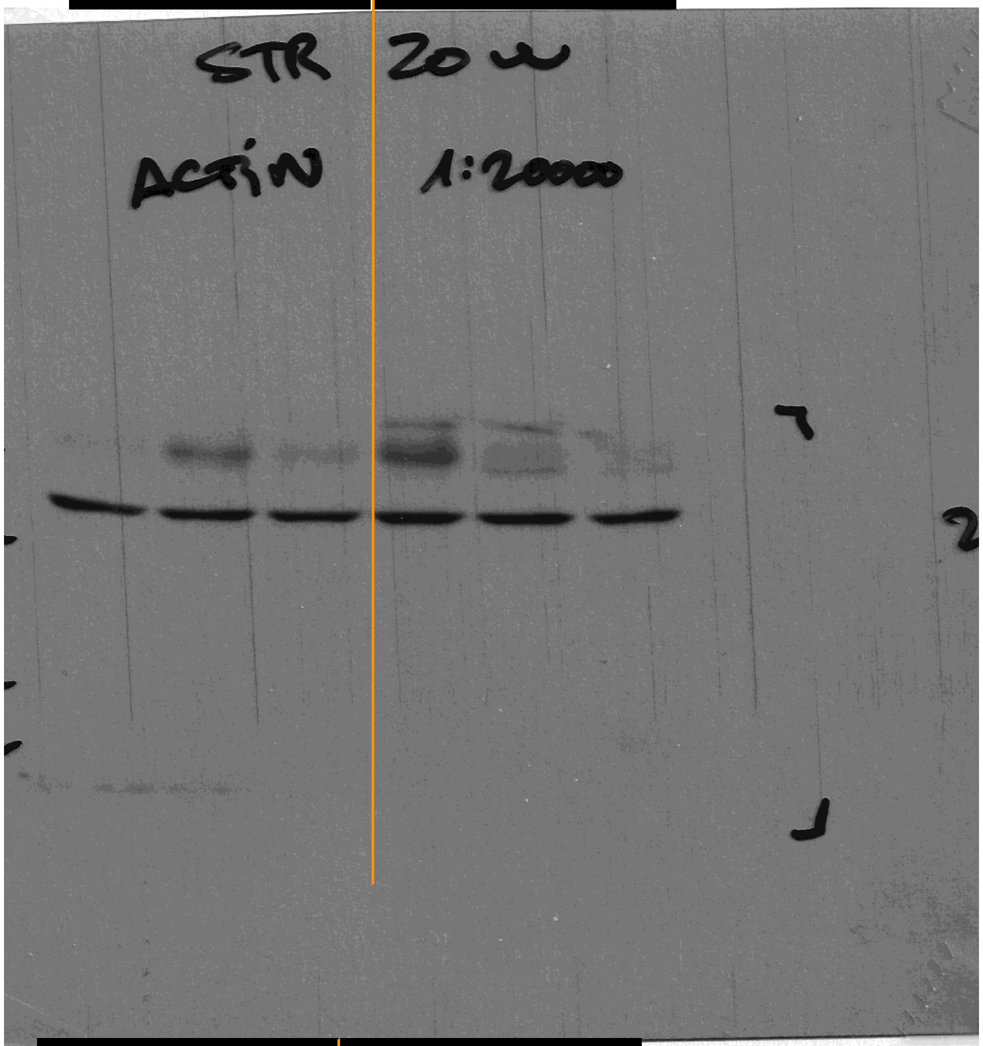

Wt	R6/1

CD200
STRIATUM
20 weeks

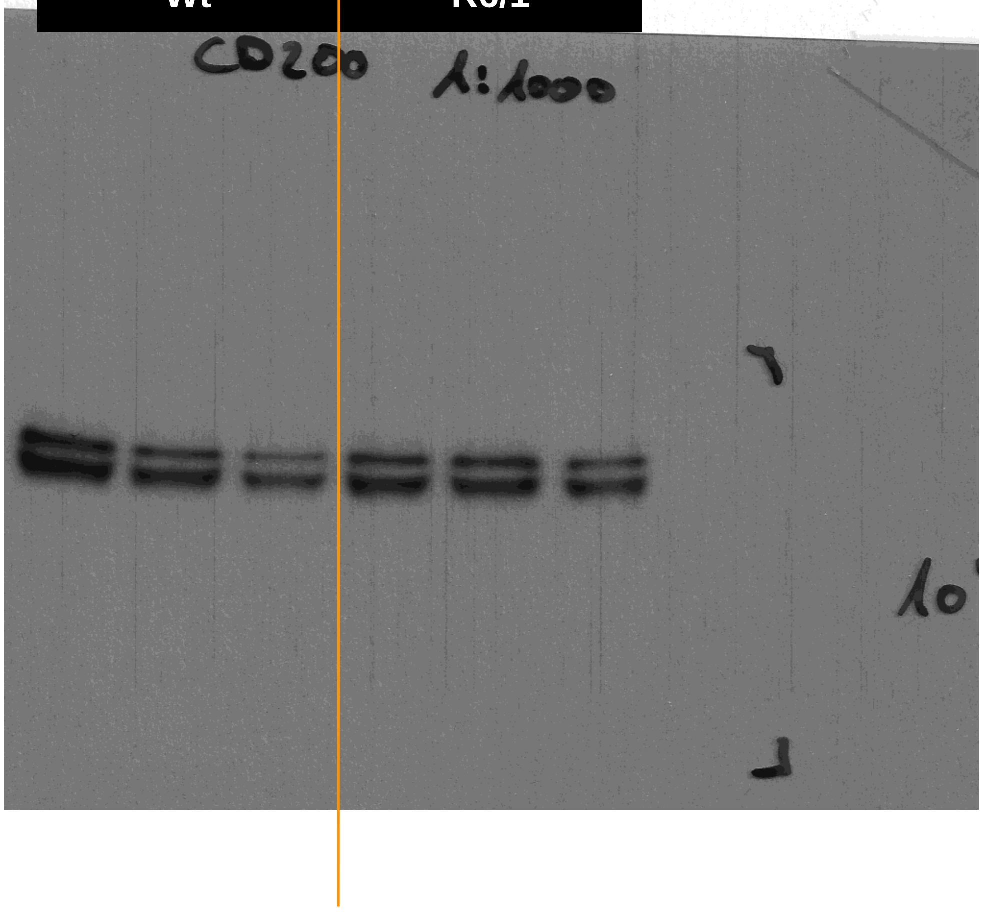

Figura 1 A: 30 weeks samples page 1/2

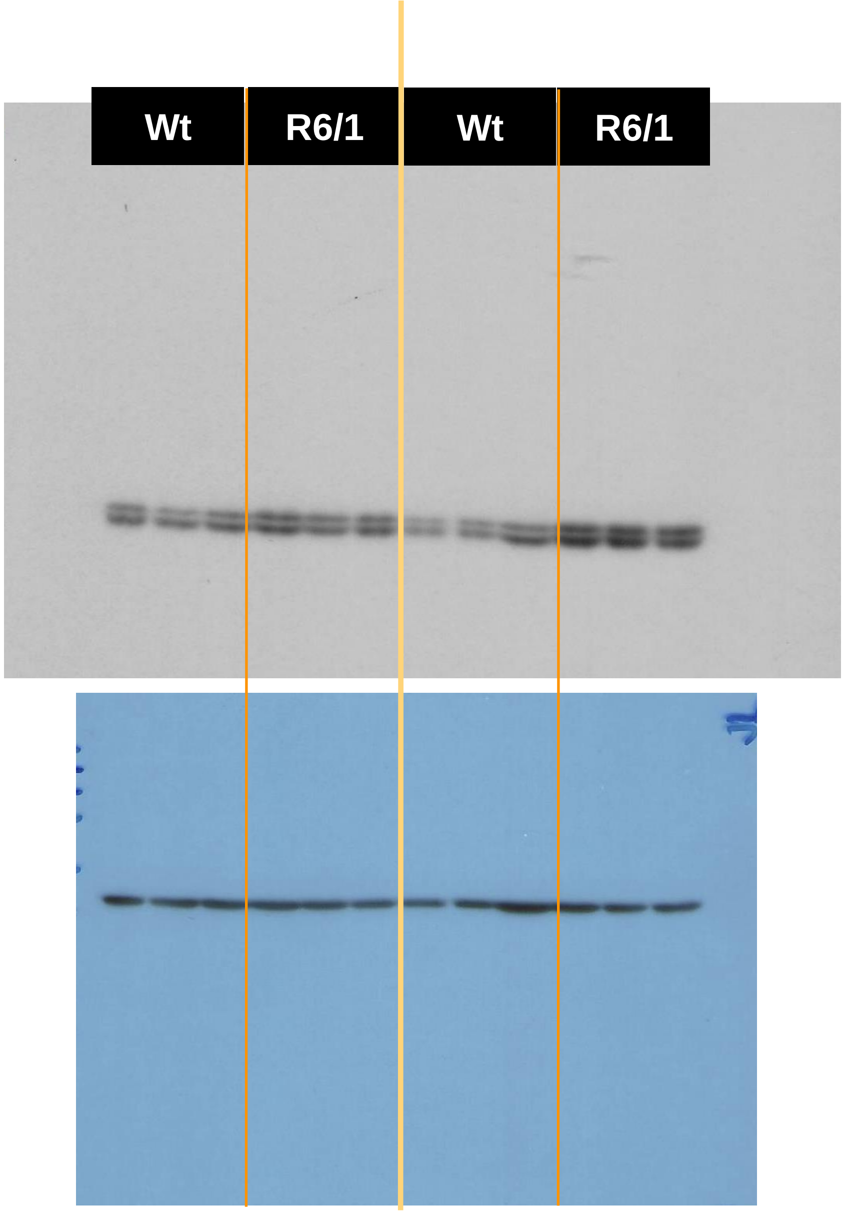

CD200
NEOCORTEX
12 - 30 weeks

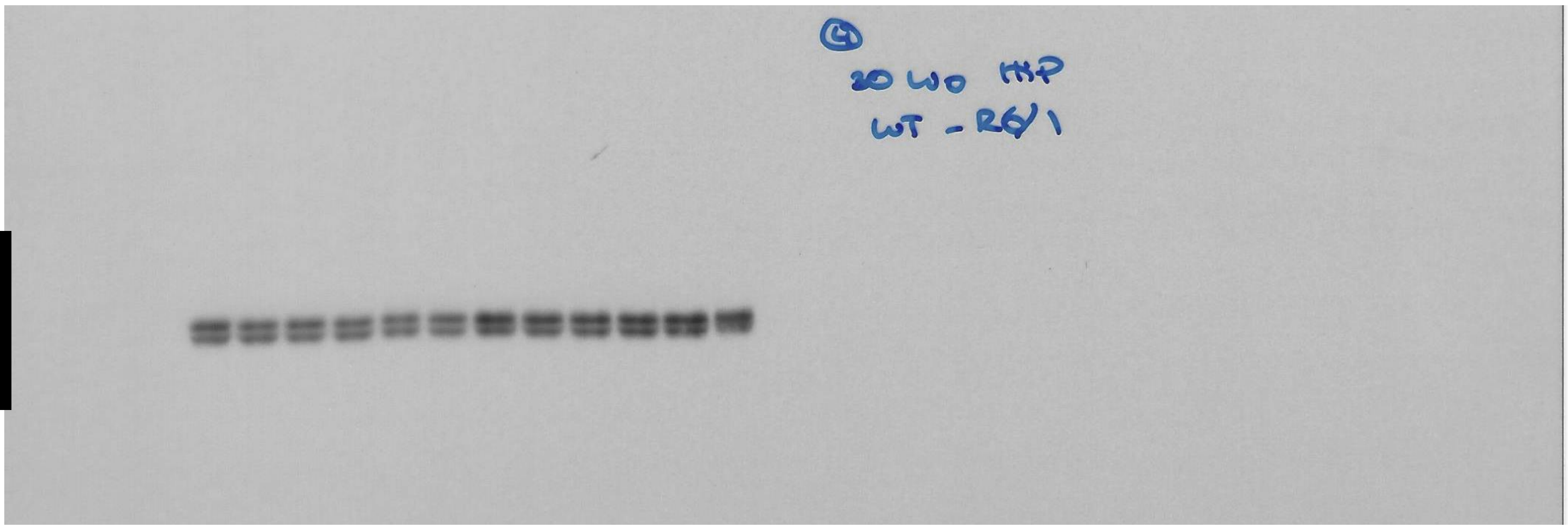

CD200
HIPPOCAMPUS
30 weeks

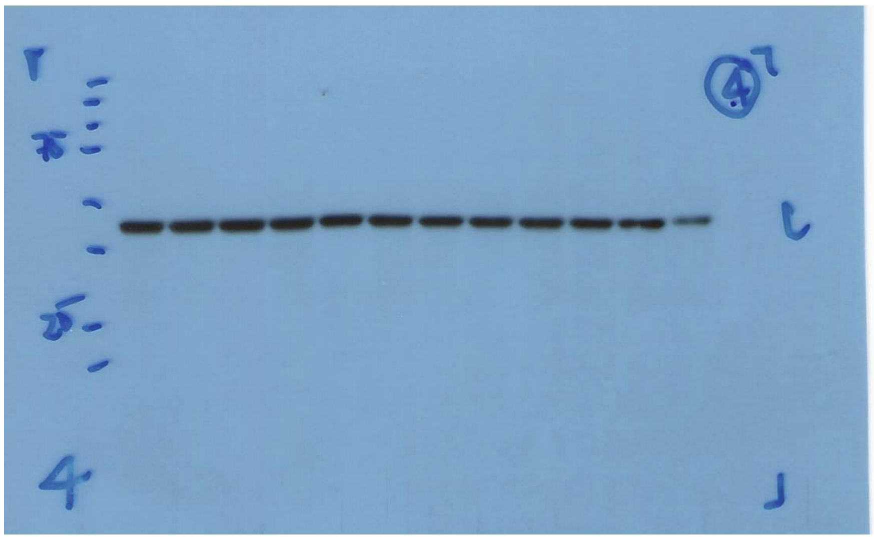

Beta-ACTIN
HIPPOCAMPUS
30 weeks

Figura 1 A: 30 weeks samples
page 2/2

CD200
STRIATUM
30 weeks

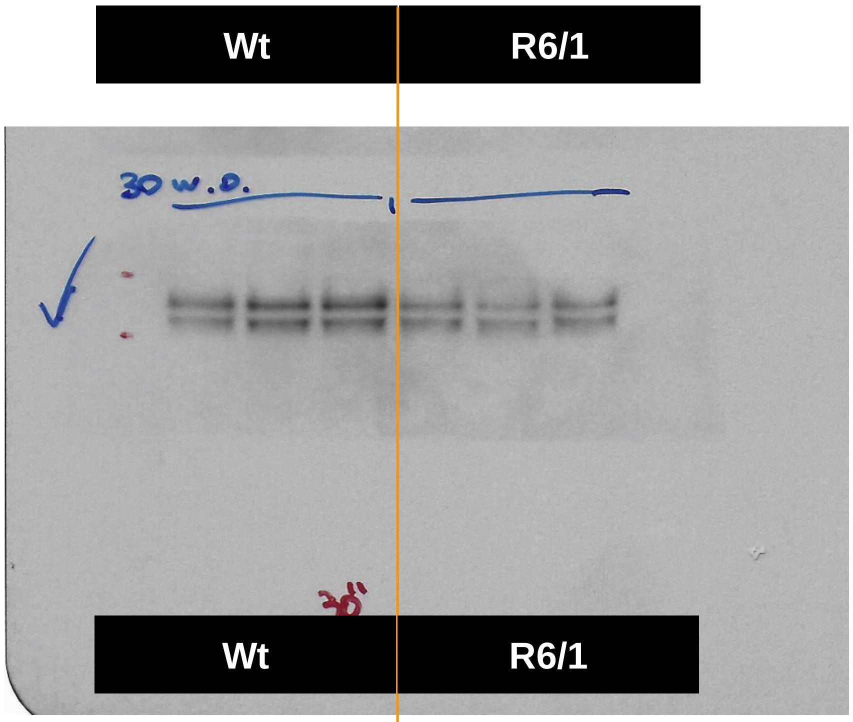

Beta-ACTIN
STRIATUM
30 weeks

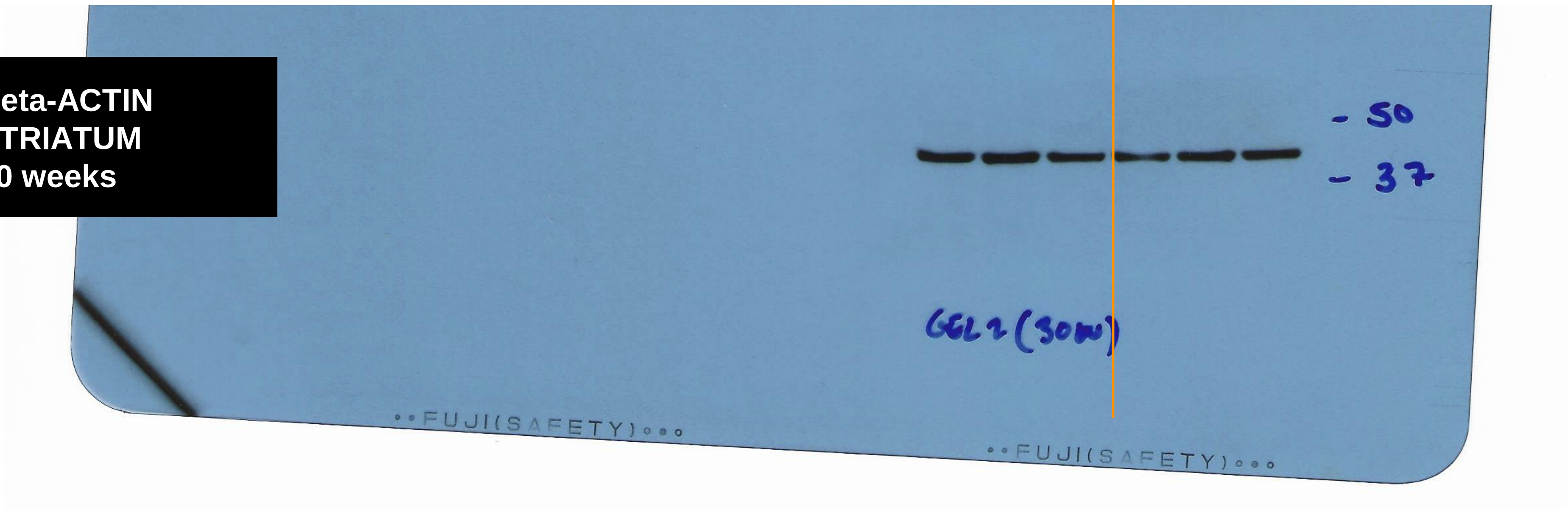

Figura 3: Knock-In 6 months
page 1/1

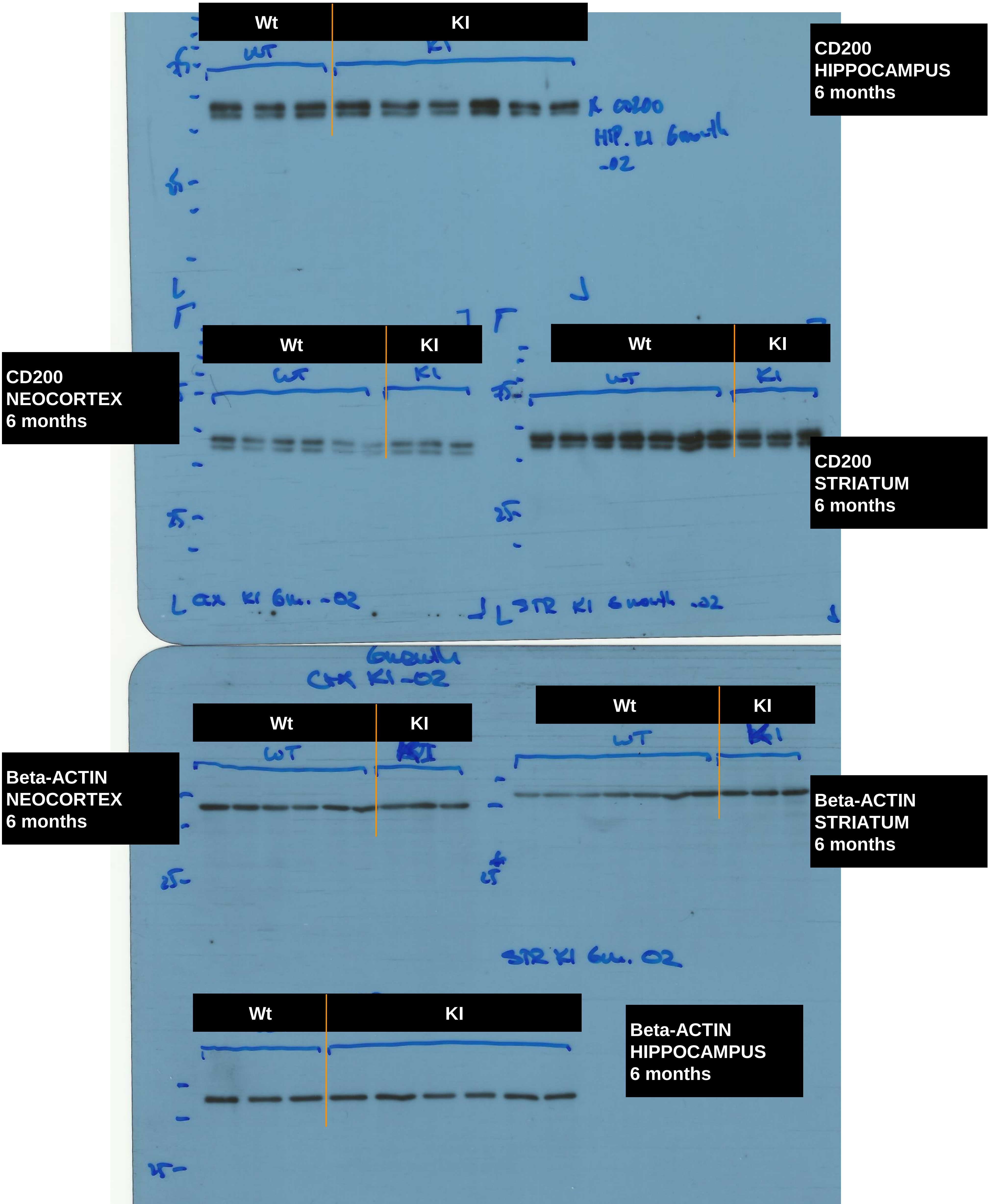

Figura 3 A and B: Knock-In 8 months page 1/2

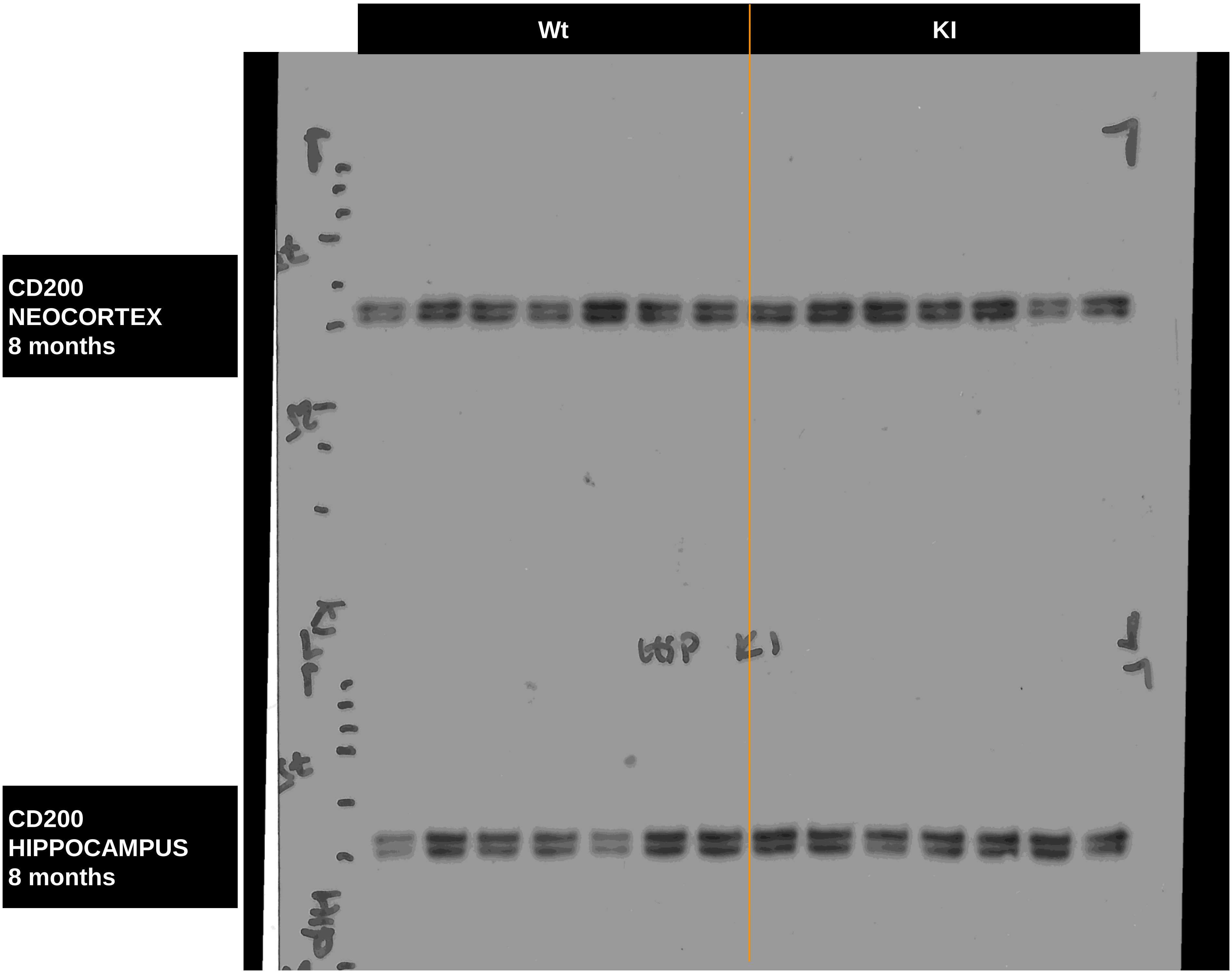

Figura 3 A and B: Knock-In 8 months page 2/2

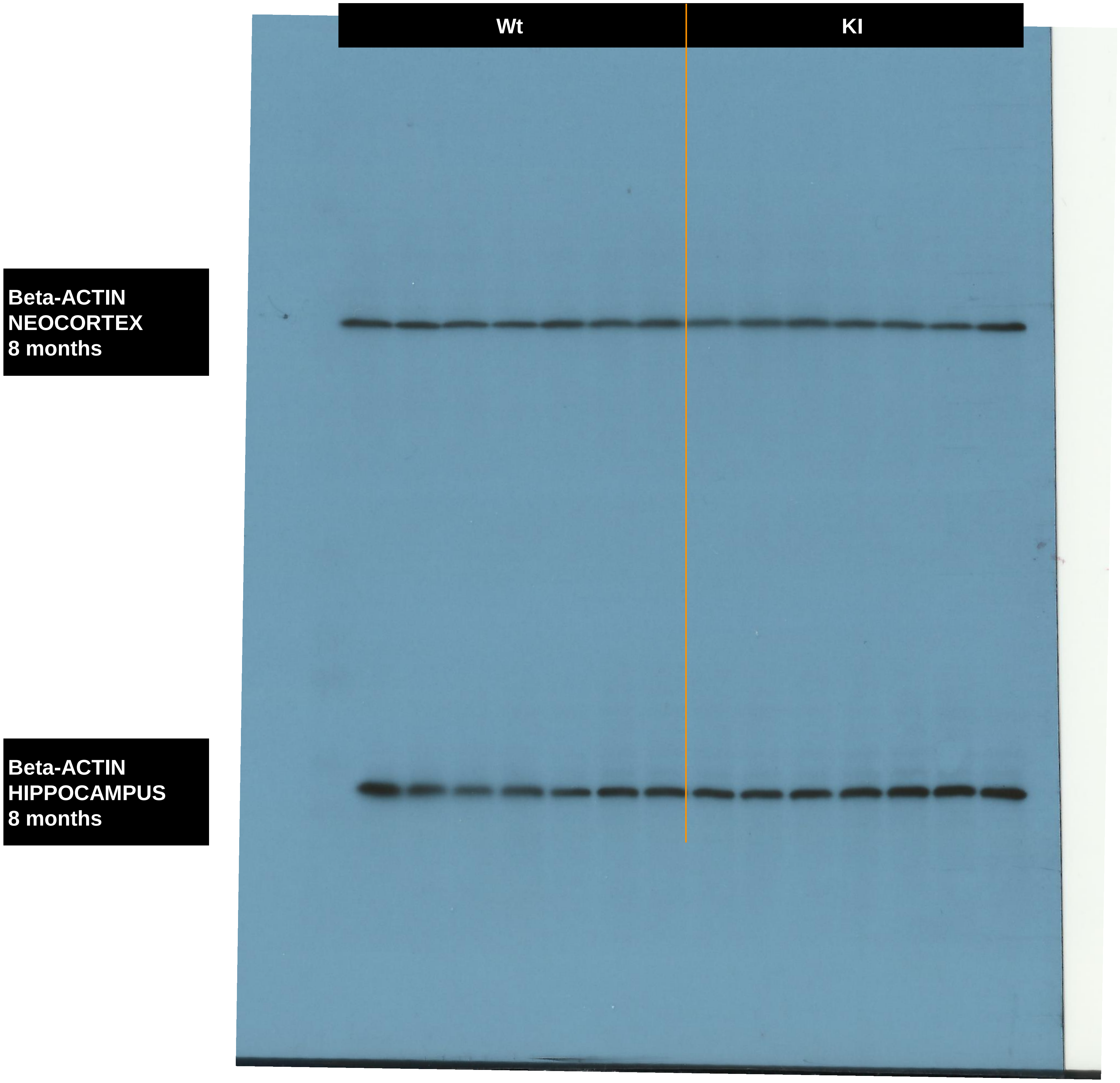

Figura 3C: Knock-In 8 months

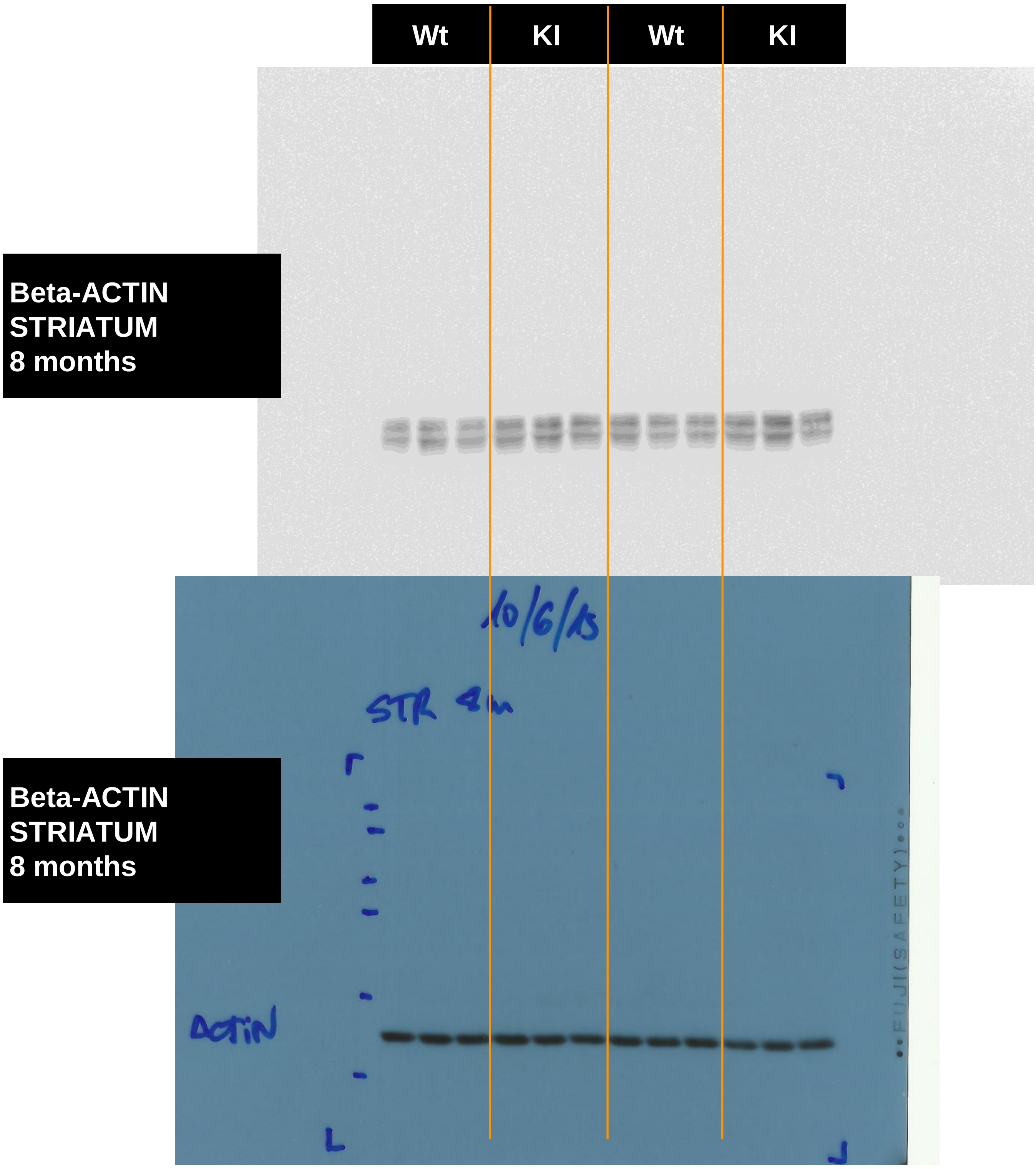

Figura 3: Knock-In 13 months
page 1/2

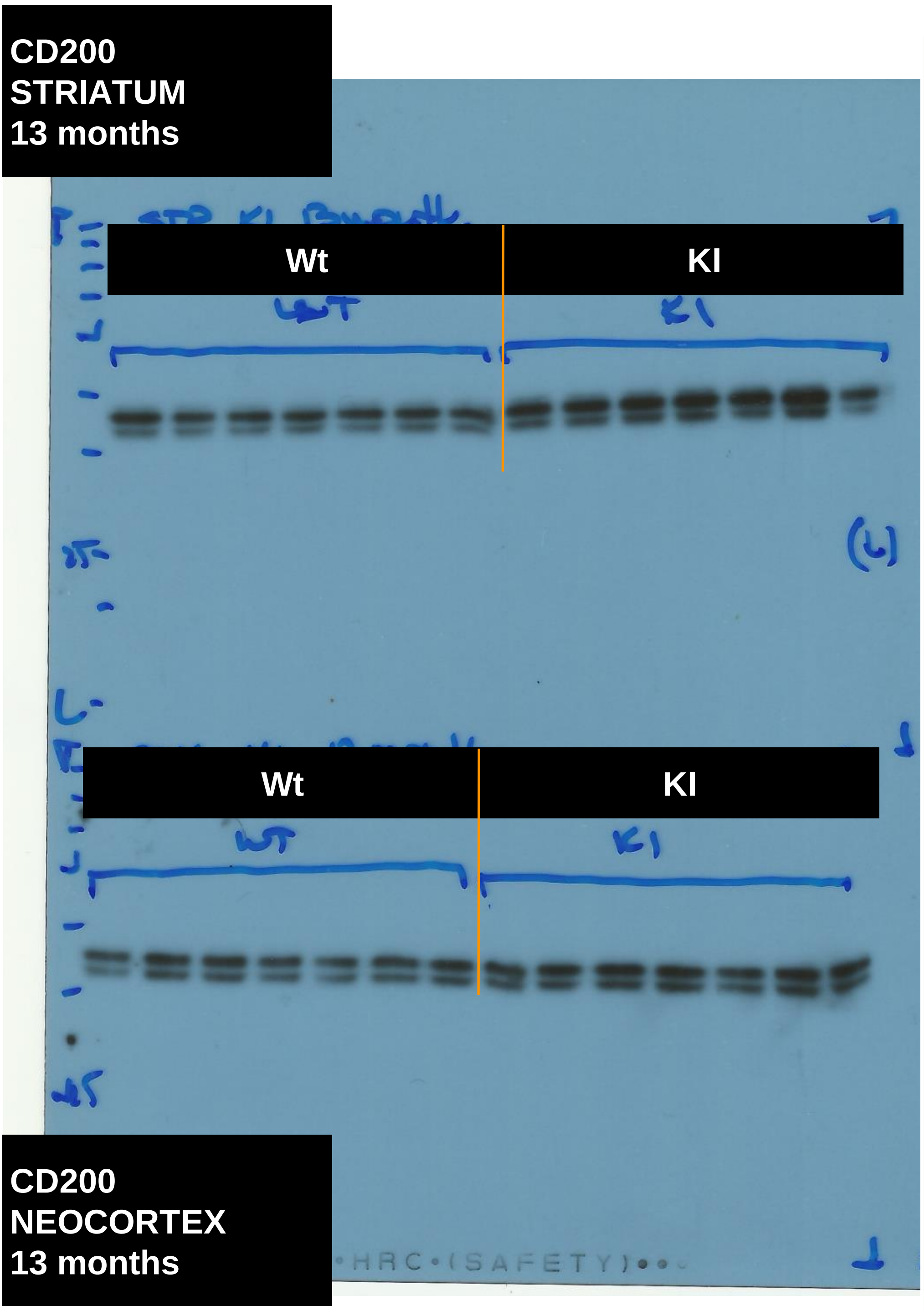

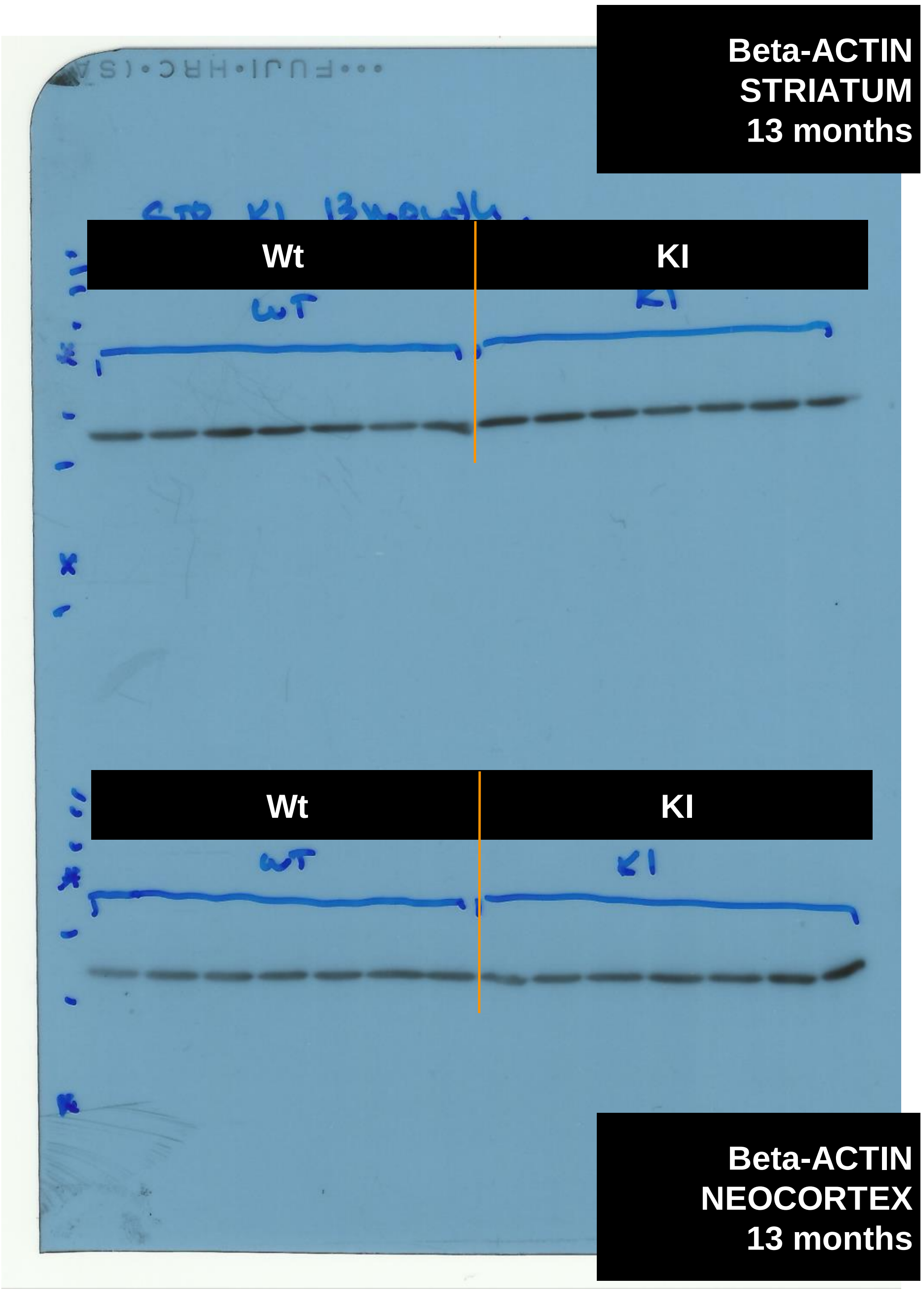

Figura 3: Knock-In 13 months
page 2/2

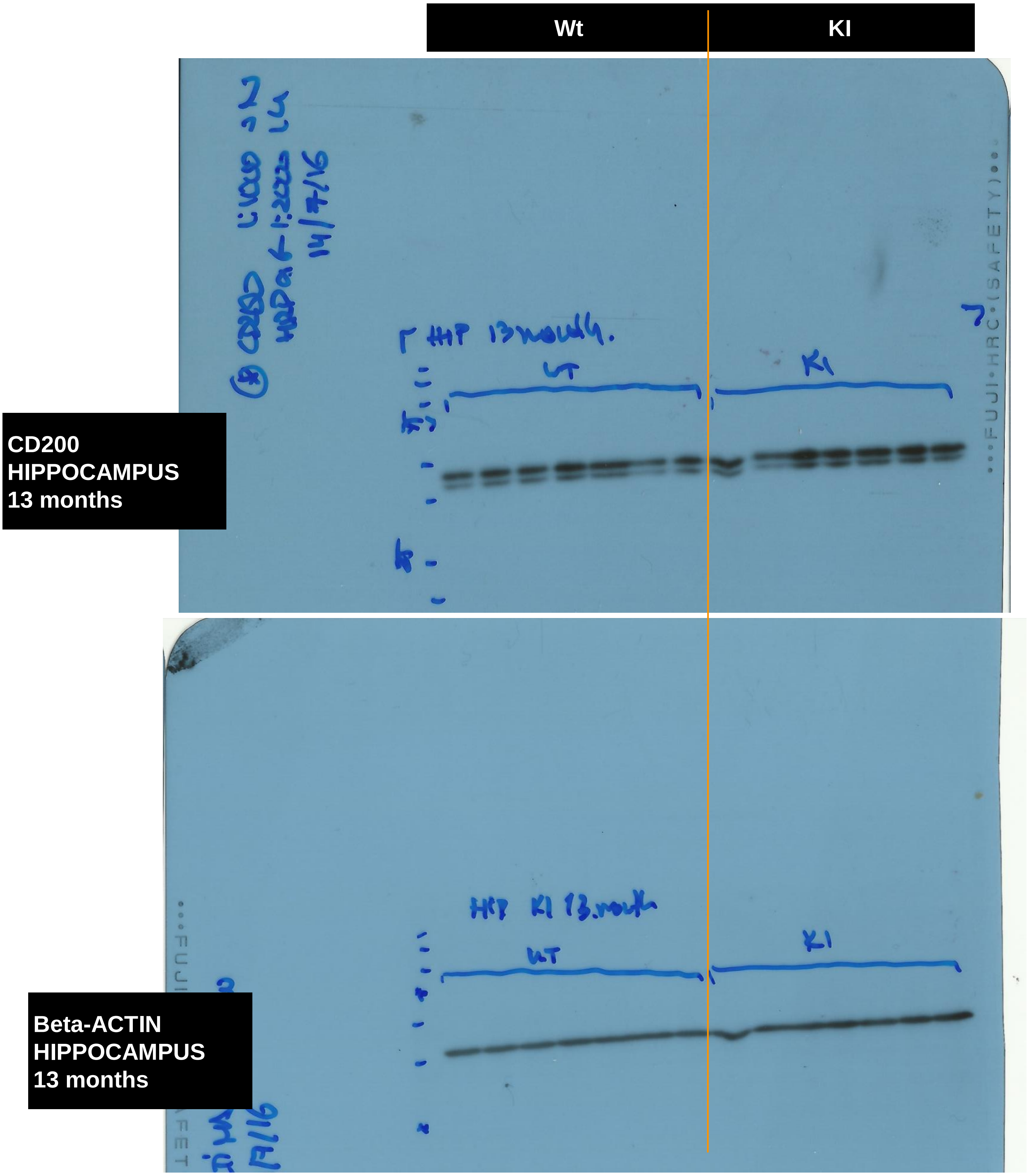

Supplement: S1 File — (PDF) [file pone.0224901.s001.pdf]
